# Limited Utility of Polymerase Chain Reaction in Induced Sputum Specimens for Determining the Causes of Childhood Pneumonia in Resource-Poor Settings: Findings From the Pneumonia Etiology Research for Child Health (PERCH) Study

**DOI:** 10.1093/cid/cix098

**Published:** 2017-05-27

**Authors:** Donald M. Thea, Phil Seidenberg, Daniel E. Park, Lawrence Mwananyanda, Wei Fu, Qiyuan Shi, Henry C. Baggett, W. Abdullah Brooks, Daniel R. Feikin, Stephen R.C. Howie, Maria Deloria Knoll, Karen L. Kotloff, Orin S. Levine, Shabir A. Madhi, Katherine L. O’Brien, J. Anthony G. Scott, Martin Antonio, Juliet O. Awori, Vicky L. Baillie, Andrea N. DeLuca, Amanda J. Driscoll, Melissa M. Higdon, Lokman Hossain, Yasmin Jahan, Ruth A. Karron, Sidi Kazungu, Mengying Li, David P. Moore, Susan C. Morpeth, Ogochukwu Ofordile, Christine Prosperi, Ornuma Sangwichian, Pongpun Sawatwong, Mamadou Sylla, Milagritos D. Tapia, Scott L. Zeger, David R. Murdoch, Laura L. Hammitt, for the PERCH Study Group, K. L. O., O. S. L., M. D. K., D. R. F., A. N. D., A. J. D., Nicholas Fancourt, W. F., L. L. H., M. M. H., E. Wangeci Kagucia, R. A. K., M. L., D. E. P., C. P., Zhenke Wu, S. L. Z., Nora L. Watson, Jane Crawley, D. R. M., B. W. A., Hubert P. Endtz, Zaman Khalequ, Doli Goswami, L. H., Y. J., Hasan Ashraf, S. R. C. H., Bernard E. Ebruke, M. A., Jessica McLellan, Eunice Machuka, Arifin Shamsul, Syed M. A. Zaman, Grant Mackenzie, J. A. G. S., J. O. A., S. C. M., Alice Kamau, S. K., Micah Silaba Ominde, K. L. K., M. D. T., Samba O. Sow, M. S., Boubou Tamboura, Uma Onwuchekwa, Nana Kourouma, Aliou Toure, S. A. M., D. P. M., Peter V. Adrian, V. L. B., Locadiah Kuwanda, Azwifarwi Mudau, Michelle J. Groome, Nasreen Mahomed, H. C. B., Somsak Thamthitiwat, Susan A. Maloney, Charatdao Bunthi, Julia Rhodes, P. S., Pasakorn Akarasewi, D. M. T., L. M., James Chipeta, P. S., James Mwansa, Somwe wa Somwe, Geoffrey Kwenda, Trevor P. Anderson, Joanne Mitchell

**Affiliations:** 1 Center for Global Health and Development, Boston University School of Public Health, Massachusetts;; 2 Department of Emergency Medicine, University of New Mexico, Albuquerque;; 3 Department of International Health, International Vaccine Access Center, Johns Hopkins Bloomberg School of Public Health, Baltimore, Maryland;; 4 Milken Institute School of Public Health, Department of Epidemiology and Biostatistics, George Washington University, DC;; 5 University Teaching Hospital, Lusaka, Zambia;; 6 Department of Rheumatology, Johns Hopkins School of Medicine, Baltimore, Maryland;; 7 Global Disease Detection Center, Thailand Ministry of Public Health-US Centers for Disease Control and Prevention Collaboration, Nonthaburi;; 8 Division of Global Health Protection, Center for Global Health, Centers for Disease Control and Prevention, Atlanta, Georgia;; 9 Department of International Health, Johns Hopkins Bloomberg School of Public Health, Baltimore, Maryland;; 10 International Centre for Diarrhoeal Disease Research, Bangladesh (icddr,b), Dhaka and Matlab;; 11 Division of Viral Diseases, National Center for Immunizations and Respiratory Diseases, Centers for Disease Control and Prevention, Atlanta, Georgia;; 12 Medical Research Council Unit, Basse, The Gambia;; 13 Department of Paediatrics University of Auckland and; 14 Centre for International Health, University of Otago, Dunedin, New Zealand;; 15 Division of Infectious Disease and Tropical Pediatrics, Department of Pediatrics, Center for Vaccine Development, Institute of Global Health, University of Maryland School of Medicine, Baltimore;; 16 Bill & Melinda Gates Foundation, Seattle, Washington;; 17 Medical Research Council: Respiratory and Meningeal Pathogens Research Unit and; 18 Department of Science and Technology/National Research Foundation: Vaccine Preventable Diseases Unit, University of the Witwatersrand, Johannesburg, South Africa;; 19 Kenya Medical Research Institute-Wellcome Trust Research Programme, Kilifi;; 20 Department of Infectious Disease Epidemiology, London School of Hygiene & Tropical Medicine and; 21 London School of Hygiene & Tropical Medicine, London, and; 22 Microbiology and Infection Unit, Warwick Medical School, University of Warwick, Coventry, United Kingdom;; 23 Department of Epidemiology,; 24 Department of International Health, Center for Immunization Research, and; 25 Department of Population, Family and Reproductive Health, Johns Hopkins Bloomberg School of Public Health, Baltimore, Maryland;; 26 Department of Paediatrics & Child Health, Chris Hani Baragwanath Academic Hospital and University of the Witwatersrand, South Africa;; 27 Microbiology Laboratory, Middlemore Hospital, Counties Manukau District Health Board, Auckland, New Zealand;; 28 Centre pour le Déloppement des Vaccins (CVD-Mali), Bamako, Mali;; 29 Department of Biostatistics, Johns Hopkins Bloomberg School of Public Health, Baltimore, Maryland;; 30 Department of Pathology, University Otago and; 31 Microbiology Unit, Canterbury Health Laboratories, Christchurch, New Zealand; 32 Johns Hopkins Bloomberg School of Public Health, Baltimore, Maryland; 33 the Emmes Corporation, Rockville, Maryland; 34 Nuffield Department of Clinical Medicine, University of Oxford, United Kingdom; 35 University of Otago, Christchurch, New Zealand; 36 icddr,b, Dhaka and Matlab, Bangladesh; 37 Medical Research Council, Basse, The Gambia; 38 KEMRI-Wellcome Trust Research Programme, Kilifi, Kenya; 39 Division of Infectious Disease and Tropical Pediatrics, Department of Pediatrics, Center for Vaccine Development, Institute of Global Health, University of Maryland School of Medicine, Baltimore, and Centre pour le Développement des Vaccins (CVD-Mali), Bamako, Mali; 40 Respiratory and Meningeal Pathogens Research Unit, University of the Witwatersrand, Johannesburg, South Africa; 41 Thailand Ministry of Public Health–US CDC Collaboration, Nonthaburi; 42 Boston University School of Public Health, Massachusetts, and University Teaching Hospital, Lusaka, Zambia; 43 Canterbury Health Laboratory, Christchurch, New Zealand

**Keywords:** induced sputum, PCR, pneumonia etiology, nasopharyngeal swab, community-acquired pneumonia.

## Abstract

**Background.:**

Sputum examination can be useful in diagnosing the cause of pneumonia in adults but is less well established in children. We sought to assess the diagnostic utility of polymerase chain reaction (PCR) for detection of respiratory viruses and bacteria in induced sputum (IS) specimens from children hospitalized with severe or very severe pneumonia.

**Methods.:**

Among children aged 1–59 months, we compared organism detection by multiplex PCR in IS and nasopharyngeal/oropharyngeal (NP/OP) specimens. To assess whether organism presence or density in IS specimens was associated with chest radiographic evidence of pneumonia (radiographic pneumonia), we compared prevalence and density in IS specimens from children with radiographic pneumonia and children with suspected pneumonia but without chest radiographic changes or clinical or laboratory findings suggestive of pneumonia (nonpneumonia group).

**Results.:**

Among 4232 cases with World Health Organization–defined severe or very severe pneumonia, we identified 1935 (45.7%) with radiographic pneumonia and 573 (13.5%) with nonpneumonia. The organism detection yield was marginally improved with IS specimens (96.2% vs 92.4% for NP/OP specimens for all viruses combined [*P* = .41]; 96.9% vs 93.3% for all bacteria combined [*P* = .01]). After accounting for presence in NP/OP specimens, no organism was detected more frequently in the IS specimens from the radiographic pneumonia compared with the nonpneumonia cases. Among high-quality IS specimens, there were no statistically significant differences in organism density, except with cytomegalovirus, for which there was a higher quantity in the IS specimens from cases with radiographic pneumonia compared with the nonpneumonia cases (median cycle threshold value, 27.9 vs 28.5, respectively; *P* = .01).

**Conclusions.:**

Using advanced molecular methods with IS specimens provided little additional diagnostic information beyond that obtained with NP/OP swab specimens.

## Summary

Among children with chest radiograph–confirmed pneumonia, polymerase chain reaction demonstrated relatively few additional pathogens in induced sputum specimens compared with nasopharyngeal/oropharyngeal specimens.

Although the World Health Organization (WHO) recommends empiric treatment for community-acquired pneumonia (CAP) in children [1], determining the causative pathogen is important if empiric therapy fails and important at a population level to guide treatment and prevention strategies. However, determining the cause of pneumonia by sampling the lung directly through bronchoscopy or transthoracic lung aspiration is possible in only a subset of cases and requires technical expertise not available in most settings [2]. 

A definitive etiologic diagnosis can be made by isolating pathogens from blood or parapneumonic fluid, but yields are low (<10%) [3]. Gram stain or culture of a sputum specimen correlates well with blood isolation of *Streptococcus pneumoniae* (57% and 79%, respectively) in adults with pneumonia [4], about 75% of whom can produce a specimen adequate for bacterial pathogen identification [5, 6]. However, there is less experience using sputum specimens in children for etiologic diagnosis because of the challenges posed by contamination with upper respiratory tract secretions and frequent colonization of the upper respiratory tract with known respiratory pathogens, such as pneumococcus [7, 8]. Although upper respiratory tract specimens (eg, nasopharyngeal [NP] swab specimens) are often used for diagnostic purposes in children with respiratory viral and some bacterial infections, there is concern whether the results reflect the cause of pneumonia [9, 10].

Examination of induced sputum (IS) specimens for suspected *Pneumocystis jirovecii* or *Mycobacterium tuberculosis* in children is useful and is now the standard of care [11, 12]. In studies of children with CAP in Finland [13, 14], New Caledonia [15], Kenya [16], and China [17], IS specimen collection was found to be well tolerated, and specimens were largely of good quality and with high frequencies of bacterial and viral putative pathogens. However, the conclusion about the significance of these pathogens as causes of pneumonia is limited because only the Kenya study included a control group.

Molecular diagnostic methods have the potential to improve our ability to detect small numbers of organisms in tissue and body fluids, thus making them an attractive potential approach for the diagnosis of pneumonia [18, 19]. Nucleic acid amplification tests can be used to detect nucleic acid from potentially all respiratory pathogens, are not dependent on viable organisms or fastidious culture conditions, and are not as affected by prior exposure to antibiotics as conventional culture methods. Using data from a large study of children hospitalized with pneumonia, we assessed the prevalence of pathogens in IS compared with nasopharyngeal/oropharyngeal (NP/OP) specimens. In addition, to determine whether the presence or quantity of respiratory pathogens detected with polymerase chain reaction (PCR) in IS specimens was associated with clinical pneumonia status, we compared hospitalized children enrolled in the Pneumonia Etiology Research for Child Health (PERCH) study who had radiographic evidence of pneumonia (radiographic pneumonia) with those who had a normal chest radiograph and a low likelihood of clinical pneumonia. The use of microbiological cultures of IS specimens for the diagnosis of childhood pneumonia is reported separately [20].

## METHODS

### Clinical Methods

The PERCH study sought to determine the cause of severe pneumonia in children aged 1–59 months living in developing areas [21]. The detailed methods of this multisite case-control study, including a description of case and control enrollment, have been published elsewhere [22, 23]. Briefly, in each of 9 sites in 7 countries (Dhaka and Matlab, Bangladesh; Basse, The Gambia; Kilifi, Kenya; Bamako, Mali; Soweto, South Africa; Sa Kaeo and Nakhon Phanom, Thailand; and Lusaka, Zambia), from August 2011 to January 2014, children presenting to study facilities with WHO-defined severe or very severe pneumonia were evaluated for enrollment using standardized criteria. Controls were randomly selected children from the community without severe or very severe pneumonia and were frequency matched for age (4 age strata) within 2 weeks of the matched case enrollment. Blood, NP/OP swab specimens, and urine were collected from both cases and controls; blood cultures and IS specimens were also collected from cases.

IS specimen collection was attempted within 24 hours of hospital admission for each PERCH case unless contraindicated. The methods for sputum induction are described elsewhere [24, 25]. In brief, participants were given a β2-agonist followed by nebulized hypertonic saline solution. A sterile closed-loop mucus extractor attached to a suction device was inserted through the nose into the nasopharynx. Suction was applied once the extractor was in the nasopharynx and was discontinued before the extractor was removed. Secretions were flushed into a collection container using 5 mL of sterile normal saline.

### Laboratory Methods

All laboratory methods were highly standardized across all study laboratories [25, 26]. Quantitative real-time PCR for respiratory pathogens was performed on IS and NP/OP specimens, as described elsewhere [27].

### Chest Radiograph

Chest radiographs from cases were classified as consolidation, other infiltrate, both consolidation and other infiltrate, normal, or uninterpretable by members of a panel of 14 radiologists and pediatricians, who were trained in the standardized interpretation of pediatric chest radiographs [28, 29].

### Study Deﬁnitions

Because the WHO definitions for severe and very severe pneumonia are not specific [30], a proportion of children enrolled as PERCH cases probably did not have true pneumonia. We therefore defined 2 subsets of PERCH cases: (1) children with radiographic evidence of pneumonia defined by the presence of consolidation and/or other infiltrate on chest radiographs, representing children who *truly* had infectious pneumonia, and (2) children with clinical and laboratory characteristics highly indicative of the *absence* of pneumonia, representing children enrolled into the case arm but with a very *low* likelihood of having infectious pneumonia (ie, nonpneumonia group; Text Box 1). Because IS specimens were not collected from controls, the nonpneumonia cases acted as “mock controls,” and this group was compared for similarity with PERCH control children.

Box 1. **Definition of Nonpneumonia (“Mock Control”) Group**

1. Enrollment into the PERCH study as a case with WHO-defined severe or very severe pneumonia *and*
2. Normal chest radiograph^a^*and*
3. Blood culture negative for pathogens *and*
4. In the *absence* of crackles:
  a. Normal respiratory rate^b^*or* no hypoxemia^c^
5. In the *presence* of crackles:
  a. Normal respiratory rate *and* no hypoxemia^c^

Hypoxemia was defined as oxygen saturation <92% (or < 90% at elevations of >1200 meters) or receipt of supplemental oxygen if a room air oxygen saturation reading was not available. A high-quality IS specimen was defined as a sputum specimen with <10 squamous epithelial cells per low-power field [25].

### Statistical Analysis

To test the hypothesis that pathogens causing pneumonia will be detected more frequently in IS than in NP/OP specimens, we compared individual paired IS and NP/OP specimen results among cases with radiographic pneumonia, using McNemar’s χ^2^ test. We calculated additional pathogen detection from the IS specimen, over and above the NP/OP specimen, as the ratio (number of infections detected in either specimen divided by number of infections detected in the NP/OP specimen) minus 1 and expressed as a percentage. The proportion of chest radiographic–positive cases with IS specimen density higher or lower than NP/OP specimen density (difference, ≥1 log_10_ copies/mL) was evaluated using the 2-sided sign test.

To assess whether pathogen detection in IS specimens (using multiplex PCR) was associated with radiographic evidence of pneumonia, we compared pathogen prevalence in the IS specimens of children with radiographic pneumonia and those without evidence of pneumonia (nonpneumonia) and calculated odds ratios and 95% confidence intervals (CIs), with and without adjustment for the presence of the pathogen on the NP/OP specimen. We compared PCR cycle threshold (Ct) values (a semiquantitative measure of pathogen load) in IS specimens from radiographic pneumonia and nonpneumonia case groups, using the Wilcoxon rank sum test. To compare demographic and baseline characteristics of individuals between study groups, we calculated frequencies for categorical variables and median values with interquartile ranges (75th centile minus 25th centile) for continuous variables. To evaluate the similarity of our mock control group (ie, nonpneumonia cases) with true control children, we compared the prevalence of organisms detected in the NP/OP specimens and calculated odds ratios and 95% CIs. Odds ratios were adjusted for potential confounding variables (eg, age, sex, site, and human immunodeficiency virus [HIV] status) where appropriate.

The majority of analyses involving IS specimens were restricted to high-quality specimens, those more likely to have originated from the lower respiratory tract [25]. The significance of differences between specimens or groups for the 28 organisms evaluated was assessed using Bonferroni correction (0.05/28), so that were differences were considered statistically significant at *P* < .002.

### Ethical Considerations

The study protocol was approved by the institutional review board or ethical review committee at each of the study site institutions and at Johns Hopkins Bloomberg School of Public Health. Parents or guardians of participants provided written informed consent.

## RESULTS

### Study Population

A total of 4232 cases with WHO-defined severe (n = 2862) or very severe (n = 1370) pneumonia and 5325 control children were enrolled during a 24-month period. IS specimens were collected from 3800 (89.8%) cases, including 94.8% (n = 2713) of those with severe pneumonia and 79.3% (n = 1087) with very severe pneumonia (Supplementary Table 1). Reasons for noncollection of IS specimens included presence of a contraindication (169 of 432; 39.1%), death (96 of 432; 22.2 %), intubation (85 of 432; 19.7%), parent refusal (14 of 432; 3.2%), and other or unknown reason (68 of 432; 15.7%). The IS specimen collection procedure was generally well tolerated, but adverse consequences were reported in 13 cases (0.34% of procedures); the most common was a transient drop in oxygen saturation [31]. Of the 4232 cases enrolled in PERCH, 1935 (45.7%) had radiographic pneumonia, 573 (13.5%) were classified as nonpneumonia, and 1724 (40.7%) fell into neither category (Table 1). This analysis is restricted to a comparison of the 1935 cases with radiographic pneumonia, 573 nonpneumonia cases, and 5325 controls.

**Table 1.** Clinical and Laboratory Features of Cases With Radiographic Pneumonia, Cases With Nonpneumonia, and Cases Excluded From the Analysis Data Set^a^

Feature	Cases, No. (%)^b^			*P* Value^c^
	Radiographic Pneumonia (n = 1935)	Nonpneumonia (n = 573)	Excluded (n = 1724)	
Age (mo), median (IQR)	8.0 (13.0)	8.0 (16.0)	7.0 (13.0)	.11
Age group				
1–11 mo	1229 (63.5)	348 (60.7)	1128 (65.4)	.23
1–5 mo	761 (39.3)	225 (39.3)	748 (43.4)	.32
6–11 mo	468 (24.2)	123 (21.5)	380 (22.0)	
12–59 mo	706 (36.5)	225 (39.3)	596 (34.6)	
Female sex	859 (44.4)	207 (36.1)	750 (43.5)	<.001
Fever^d^	1614 (83.4)	463 (80.8)	1373 (79.6)	.15
WBC abnormality^e^	932 (51.2)	202 (37.0)	729 (45.2)	<.001
Malaria slide positive	20 (1.0)	44 (7.7)	29 (1.7)	<.001
HIV infection	166 (8.6)	8 (1.4)	77 (4.5)	<.001
High-quality IS specimen^f^	1166 (66.6)	398 (74.8)	1049 (69.2)	<.001
Prior use of antibiotics^g^	1393 (79.5)	365 (68.6)	1122 (74.0)	<.001
Tachypnea^h^	1629 (84.4)	293 (51.1)	1545 (89.7)	
Hypoxemia^i^	862 (44.6)	32 (5.6)	694 (40.4)	
Oxygen requirement	271 (14.0)	13 (2.3)	233 (13.5)	
Crackles at chest auscultation	1326 (69.0)	103 (18.0)	1287 (75.0)	
Bacteremia^j^	73 (3.8)	0 (0.0)	74 (4.4)	

Abbreviations: CXR, chest radiograph; HIV, human immunodeficiency virus; IQR, interquartile range (75th centile minus 25th centile); IS, induced sputum; WBC, white blood cell.

^a^Radiographic pneumonia was defined as radiographic evidence of pneumonia (consolidation and/or other infiltrates); nonpneumonia, as normal chest radiograph, blood culture negative for pathogens, and either (1) a normal respiratory rate *or* no hypoxemia in the absence of crackles or (2) a normal respiratory rate *and* no hypoxemia in the presence of crackles. Excluded cases were those who did not meet either definition.

^b^Data represent No. (%) of cases unless otherwise specified.

^c^ *P* values for comparison between radiographic pneumonia and nonpneumonia groups (χ^2^ or Wilcoxon test); values are not presented for variables used to define nonpneumonia status.

^d^Fever was defined as documented temperature of ≥38.0°C or fever in the past 48 hours as reported by caregiver.

^e^WBC count ≥15 or ≥13 × 10^9^/L for children aged 1–11 or 12–59 months, respectively, or <5 × 10^9^/L for children of any age.

^f^High-quality IS specimens were defined as <10 squamous epithelial cells per low-power field.

^g^Prior use of antibiotics was defined as positive serum bioassay results, antibiotic administration at the referral facility, or antibiotic administration before IS specimen collection at the study facility.

^h^Tachypnea was defined as ≥60, ≥50, or ≥ 40 breaths per minute for children aged <2, 2–11, or 12–59 months, respectively.

^i^Hypoxemia was defined as (1) a room air pulse oximetric reading indicated oxygen saturation <90% at the 2 sites at elevation (Zambia and South Africa) or <92% at all other sites or (2) receipt of supplemental oxygen if a room air oxygen saturation reading was not available.

^j^Microbiologically confirmed isolation of bacterial organism from sterile site (eg, blood or parapneumonic fluid).

The proportion cases with radiographic pneumonia varied by site, from 31% at Matlab, Bangladesh, to 60% at Dhaka, Bangladesh, and Soweto, South Africa (Table 2). The proportion of nonpneumonia cases among those enrolled varied by site from none in Dhaka, Bangladesh and 4.5% of cases in Soweto, South Africa, to 31% enrolled from Kilifi, Kenya (Table 2). Compared with nonpneumonia cases, cases with radiographic pneumonia were more likely to be female (44% vs 36%; *P* < .001), have an abnormally high or low white blood cell count (51% vs 37%; *P* < .001), or be HIV positive (9% vs 1%; *P* < .001); malaria was more common in the nonpneumonia group (8% vs 1%; *P* < .001) (Table 1). 

**Table 2.** Characteristics of Radiographic Pneumonia and Nonpneumonia Groups by Site^a^

Characteristic	Cases, No. (%)^b^															
	Kilifi, Kenya (n = 634)		Basse, The Gambia (n = 638)		Bamako, Mali (n = 674)		Lusaka, Zambia (n = 617)		Soweto, South Africa (n = 920)		Nakhon Phanom and Sa Kaeo, Thailand (n = 224)		Matlab, Bangladesh (n = 327)		Dhaka, Bangladesh (n = 198)	
	**RP**	**Non-RP**	**RP**	**Non-RP**	**RP**	**Non-RP**	**RP**	**Non-RP**	**RP**	**Non-RP**	**RP**	**Non-RP**	**RP**	**Non-RP**	**RP**	**Non-RP**
All cases	284 (44.8)	195 (30.8)	290 (45.5)	101 (15.8)	253 (37.5)	100 (14.8)	266 (43.1)	67 (10.9)	524 (60.0)	41 (4.5)	99 (44.2)	31 (13.8)	101 (30.9)	38 (11.6)	118 (60.0)	0 (0.0)
Age (mo), median (IQR)	10.0 (14.0)^c^	13.0 (19.0)	8.5 (13.0)	7.0 (17.0)	8.0 (10.0)	8.5 (12.5)	5.0 (9.0)	4.0 (8.0)	6.0 (10.0)	4.0 (6.0)	15.0 (21.0)	13.0 (16.0)	12.0 (17.0)^c^	7.0 (21.0)	12.0 (14.0)	0 (0.0)
Age group																
1–11 mo	156 (54.9)	91 (46.7)	180 (62.1)	66 (65.3)	163 (64.4)	64 (64.0)	200 (75.2)	57 (85.1)	392 (74.8)	32 (78.0)	38 (38.4)	14 (45.2)	47 (46.5)	24 (63.2)	53 (44.9)	0 (0.0)
1–5 mo	90 (31.7)	55 (28.2)	109 (37.6)	45 (44.6)	102 (40.3)	36 (36.0)	137 (51.5)	42 (62.7)	254 (48.5)	24 (58.5)	19 (19.2)	6 (19.4)	25 (24.8)	17 (44.7)	25 (21.2)	0 (0.0)
6–11 mo	66 (23.2)	36 (18.5)	71 (24.5)	21 (20.8)	61 (24.1)	28 (28.0)	63 (23.7)	15 (22.4)	138 (26.3)	8 (19.5)	19 (19.2)	8 (25.8)	22 (21.8)	7 (18.4)	28 (23.7)	0 (0.0)
12–59 mo	128 (45.1)	104 (53.3)	110 (37.9)	35 (34.7)	90 (35.6)	36 (36.0)	66 (24.8)	10 (14.9)	132 (25.2)	9 (22.0)	61 (61.6)	17 (54.8)	54 (53.5)	14 (36.8)	65 (55.1)	0 (0.0)
Bacteremia^d^	9 (3.2)	0 (0.0)	14 (4.9)	0 (0.0)	16 (6.3)	0 (0.0)	14 (5.3)	0 (0.0)	15 (2.9)	0 (0.0)	3 (3.0)	0 (0.0)	0 (0.0)	0 (0.0)	2 (1.7)	0 (0.0)
Tachypnea^e^	224 (78.9)	87 (44.6)	264 (91.0)	54 (53.5)	215 (85.0)	66 (66.0)	245 (92.5)	40 (62.5)	396 (77.6)	11 (26.8)	75 (80.6)	18 (58.1)	93 (92.1)	17 (44.7)	117 (99.2)	0 (0.0)
Hypoxemia^f^	107 (37.8)	12 (6.2)	33 (11.4)	0 (0.0)	140 (55.3)	5 (5.0)	133 (50.2)	4 (6.0)	398 (76.1)	11 (27.5)	29 (29.3)	0 (0.0)	3 (3.0)	0 (0.0)	19 (16.1)	0 (0.0)
Crackles	134 (47.3)	16 (8.2)	234 (81.0)	30 (29.7)	186 (73.5)	15 (15.0)	147 (55.3)	12 (17.9)	347 (67.8)	9 (22.0)	68 (68.7)	3 (9.7)	93 (92.1)	18 (47.4)	117 (99.2)	0 (0.0)
High-quality IS specimen^g^	233 (86.9)	157 (85.8)	197 (74.3)	67 (69.1)	126 (59.4)	50 (56.8)	194 (82.2)	48 (81.4)	202 (43.2)	22 (56.4)	72 (90.0)	26 (96.3)	69 (68.3)	28 (73.7)	73 (61.9)	0 (0.0)
Prior use of antibiotics^h^	252 (93.7)^c^	158 (86.3)	60 (22.6)	23 (23.7)	176 (83.0)^c^	55 (62.5)	236 (98.7)	58 (96.7)	434 (92.7)	37 (94.9)	74 (92.5)^c^	21 (77.8)	43 (42.6)	13 (34.2)	118 (100)	0 (0.0)

Abbreviations: IQR, interquartile range (75th centile minus 25th centile); IS, induced sputum; Non-RP, non-radiographic pneumonia; RP, radiographic pneumonia.

^a^Radiographic pneumonia was defined as radiographic evidence of pneumonia (consolidation and/or other infiltrates); nonpneumonia, as normal chest radiograph, blood culture negative for pathogens, and either (1) a normal respiratory rate *or* no hypoxemia in the absence of crackles or (2) a normal respiratory rate *and* no hypoxemia in the presence of crackles.

^b^Percentages for radiographic pneumonia and nonpneumonia are based on total number of cases at the site; percentages for characteristics based on total number indicated in corresponding column.

^c^Significant difference between radiographic pneumonia and nonpneumonia groups at the site (not reported for bacteremia, tachypnea, hypoxemia, or crackles because they are part of the nonpneumonia definition).

^d^Microbiologically confirmed isolation of bacterial organism from sterile site (eg, blood or parapneumonic fluid).

^e^Tachypnea defined as ≥60, ≥50, or ≥ 40 breaths per minute for children aged <2, 2–11, or 12–59 months, respectively.

^f^Hypoxemia was defined as (1) a room air pulse oximetric reading indicated oxygen saturation <90% at the 2 sites at elevation (Zambia and South Africa) or <92% at all other sites or (2) receipt of supplemental oxygen if a room air oxygen saturation reading was not available.

^g^High-quality IS specimens were defined as <10 squamous epithelial cells per low-power field.

^h^Prior use of antibiotics defined as positive serum bioassay results, antibiotic administration at the referral facility, antibiotic administration before IS specimen collection at the study facility.

Characteristics used to distinguish radiographic pneumonia from nonpneumonia cases, such as hypoxemia (45% vs 6%), tachypnea for age (84% vs 51%), and the presence of crackles (69% vs 18%) differed as expected. Cases in the nonpneumonia group were more likely to produce high-quality sputum specimens (75% vs 67%; *P* < .001). The low number of cases classified as nonpneumonia in South Africa reflects the standard clinical practice at that site to administer oxygen to all children with a diagnosis of pneumonia (often without obtaining a room air oxygen saturation measurement), which led to a high percentage (75%) of cases meeting the study definition of hypoxemia. No cases met the definition of nonpneumonia at the Dhaka, Bangladesh site; 40% had a normal chest radiograph but all had other respiratory findings that excluded them from nonpneumonia status (Text Box 1).

### Comparison of NP/OP and IS Specimen Findings

To determine the concordance of pathogen detection rates between NP/OP and IS specimens, and the added yield from testing IS specimens in addition to NP/OP specimens, we analyzed NP/OP swabs and IS specimens collected from the 1692 cases admitted to the hospital with radiographic pneumonia from whom paired specimens were available. Of these 1692, 99% had 1 or more pathogens identified on IS PCR; the median number of pathogens identified on PCR of IS or NP/OP was 4. Among those with paired specimens, high quality IS specimens were available in 1114 (65.8%). Using both NP/OP and high-quality IS specimens, bacteria were detected in 1080 (96.9%) specimens and viruses in 1072 (96.2%) specimens. Overall, the IS specimens increased the number of cases with a virus, bacteria, or any organism detected by 4.1%, 3.9%, and 1.3%, respectively (Table 3). Restricting this analysis to cases <6 months of age slightly reduced the added benefit (data not shown).

**Table 3.** PCR Pathogen Detection in Paired NP/OP Swab and IS Specimens From 1114 Hospitalized Children (Aged 1–59 Months) With Radiographic Pneumonia

Pathogen	Cases, No. (%)			Odds Ratio (95% CI)^b^	P Value^b^	Increase With Use of IS Specimens (95% CI), %^c^	Agreement Between NP/OP and IS Specimens, %
	Both NP/OP and IS Specimens Positive^a^	Only NP/OP Specimens Postitive	Only IS Specimens Positive				
*Bordetella pertussis*	7 (0.6)	1 (0.1)	3 (0.3)	3.00 (.95–9.52)	.63	37.5 (7.9–154.3)	99.6
*Chlamydophila pneumoniae*	7 (0.6)	8 (0.7)	5 (0.5)	0.63 (.35–1.11)	.58	33.3 (10.6–97.7)	98.8
*Haemophilus influenzae*	527 (47.7)	116 (10.5)	66 (6.0)	0.57 (.49–.66)	< .001^d^	10.3 (7.9–13.3)	83.5
*H. influenzae* type b	16 (1.5)	9 (0.8)	6 (0.5)	0.67 (.39–1.13)	.61	24.0 (8.8–61.4)	98.6
*Moraxella catarrhalis*	623 (56.4)	125 (11.3)	36 (3.3)	0.29 (.24–.35)	<.001^d^	4.8 (3.4–6.8)	85.4
*Mycoplasma pneumoniae*	14 (1.3)	4 (0.4)	12 (1.1)	3.00 (1.68–5.34)	.08	66.7 (30.2–145.4)	98.5
*Pneumocystis jirovecii*	65 (5.9)	31 (2.8)	29 (2.6)	0.94 (.72–1.21)	.90	30.2 (19.5–46.5)	94.6
*Staphylococcus aureus*	96 (8.7)	66 (6.0)	42 (3.8)	0.64 (.52–.78)	.03	25.9 (18.2–36.8)	90.2
*Streptococcus pneumoniae*	741 (67.1)	77 (7.0)	40 (3.6)	0.52 (.43–.63)	<.001^d^	4.9 (3.5–6.8)	89.4
*Salmonella* species	3 (0.3)	8 (0.7)	9 (0.8)	1.13 (.69–1.83)	>.99	81.8 (31.3–212.0)	98.5
Adenovirus	89 (8.1)	30 (2.7)	54 (4.9)	1.80 (1.43–2.26)	.01	45.4 (32.5–63.3)	92.3
Bocavirus	76 (6.9)	71 (6.5)	79 (7.2)	1.11 (.94–1.31)	.57	53.7 (40.5–71.2)	86.3
CMV	476 (43.4)	107 (9.8)	84 (7.7)	0.79 (.68–.91)	.11	14.4 (11.4–18.2)	82.6
HCoV 229E	9 (0.8)	6 (0.5)	5 (0.5)	0.83 (.45–1.53)	>.99	33.3 (10.6–97.7)	99.0
HCoV OC43	17 (1.6)	4 (0.4)	14 (1.3)	3.50 (1.99–6.17)	.03	66.7 (32.2–136.9)	98.4
HCoV NL63	13 (1.2)	5 (0.5)	5 (0.5)	1.00 (.53–1.88)	>.99	27.8 (9.0–79.2)	99.1
HCoV HKU1	16 (1.5)	8 (0.7)	6 (0.5)	0.75 (.44–1.29)	.79	25.0 (9.2–64.3)	98.7
HMPV	89 (8.1)	28 (2.6)	39 (3.6)	1.39 (1.09–1.78)	.22	33.3 (22.8–48.5)	93.9
Influenza A	34 (3.1)	7 (0.6)	5 (0.5)	0.71 (.4–1.28)	.77	12.2 (4.2–32.2)	98.9
Influenza B	12 (1.1)	0 (0.0)	3 (0.3)	N/A	.25	25.0 (5.6–94.7)	99.7
Influenza C	4 (0.4)	4 (0.4)	1 (0.1)	0.25 (.08–.76)	.38	12.5 (.6–97.4)	99.5
Parainfluenza 1	52 (4.8)	5 (0.5)	31 (2.8)	6.20 (3.83–10.04)	<.001^e^	54.4 (34.3–85.9)	96.7
Parainfluenza 2	7 (0.6)	7 (0.6)	11 (1.0)	1.57 (.97–2.55)	.48	78.6 (33.4–183.5)	98.4
Parainfluenza 3	55 (5.0)	11 (1.0)	19 (1.7)	1.73 (1.18–2.52)	.20	28.8 (16.7–49.1)	97.3
Parainfluenza 4	20 (1.8)	13 (1.2)	6 (0.5)	0.46 (.28–.76)	.17	18.2 (6.9–45.4)	98.3
PV/EV	51 (4.6)	33 (3.0)	55 (5.0)	1.67 (1.34–2.08)	.02	65.5 (46.0–93.2)	92.0
Rhinovirus	164 (15.0)	76 (6.9)	72 (6.6)	0.95 (.8–1.12)	.81	30.0 (22.8–39.3)	86.5
RSV	246 (22.4)	50 (4.6)	27 (2.5)	0.54 (.43–.69)	.01	9.1 (6.0–13.7)	93.0
Any bacteria	1019 (91.4)	20 (1.8)	41 (3.7)	2.05 (1.56–2.69)	.01	3.9 (2.9–5.4)	94.5
Any virus	979 (87.8)	51 (4.6)	42 (3.8)	0.82 (.67–1.01)	.41	4.1 (3.0–5.6)	91.7
Any pathogen	1089 (97.7)	6 (0.5)	14 (1.3)	2.33 (1.43–3.8)	.12	1.3 (.7–2.2)	98.2

Abbreviations: CI, confidence interval; CMV, cytomegalovirus; HCoV, human coronavirus; HMPV, human metapneumovirus A/B; IS, induced sputum; NP/OP, nasopharyngeal/oropharyngeal; PCR, polymerase chain reaction; PV/EV, parechovirus/enterovirus; RSV, respiratory syncytial virus.

^a^Calculated among cases with radiographic pneumonia and available NP/OP specimens and high-quality IS specimens for each pathogen (n = 1114); some cases were missing data for certain pathogens (≤20 cases per pathogen). Radiographic evidence of pneumonia was defined as consolidation and/or other infiltrates.

^b^Odds ratio and *P* values were obtained with McNemar’s χ^2^ test. Odds ratios were calculated as the ratio of the discordant pairs (results for IS positive and NP/OP negative/results for NP/OP positive and IS negative).

^c^Percentage increase calculated as ratio (No. of infections detected with either specimen/No. of infections detected with NP/OP specimen) minus 1, expressed as a percentage.

^d^The presence in NP/OP specimens alone is significantly greater than that in IS specimens alone (*P* < .002).

^e^The presence in IS specimens alone is significantly greater than that in NP/OP specimens alone (*P* < .002).

Among cases with radiographic pneumonia, pathogen detection by PCR was similar for high-quality IS specimens and NP/OP specimens for most pathogens; this is reflected by the moderate-to-high agreement between NP/OP and IS specimens (Table 3). A similar pattern was also observed comparing high-quality IS and NP/OP specimens from nonpneumonia cases (Supplementary Table 2). The added yield of an IS specimen for identifying a pathogen in cases with radiographic pneumonia varied by organism from 4.8% (95% CI, 3.4%–6.8%; *Moraxella catarrhalis*) to 81.8% (31.3%–212.0%; *Salmonella* species). Because the added yield calculation can be misleading with small sample sizes (eg, the 81.8% for *Salmonella* species reflects a difference of 8 vs 9 detections in NP/OP vs IS specimens), it is useful to visualize the pattern of detection of different pathogens by different specimen types. 

**Figure 1.**
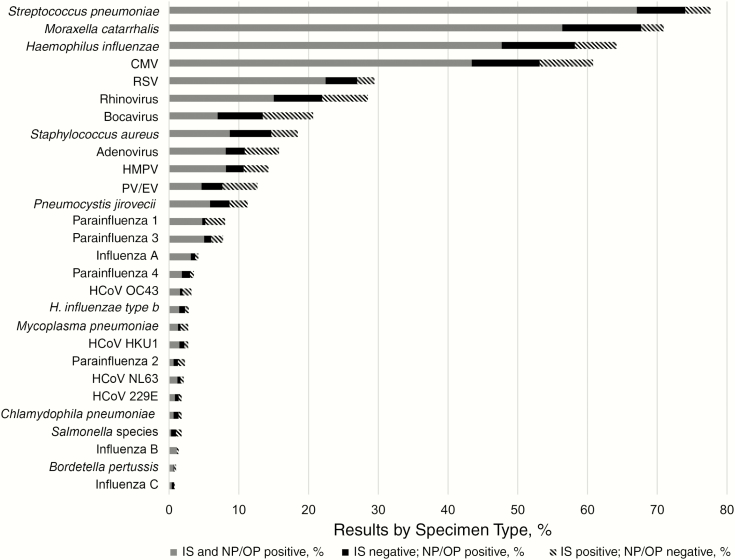
Multiplex polymerase chain reaction (PCR) pathogen detection in paired nasopharyngeal/oropharyngeal (NP/OP) swab and induced sputum (IS) specimens from 1114 children aged 1–59 months, hospitalized with radiographic evidence of pneumonia (consolidation and/or other infiltrates) and with a high-quality IS specimen available. Gray bars represent pathogen detected in both IS and NP/OP specimens; black bars, pathogen detected in NP/OP but not IS specimens; and hatched bars, pathogen detected in IS but not NP/OP specimens. High-quality IS specimens were defined as <10 squamous epithelial cells per low-power field; radiographic evidence of pneumonia was defined as consolidation and/or other infiltrates. Abbreviations: CMV, cytomegalovirus; HCoV, human coronavirus; HMPV, human metapneumovirus A/B; PV/EV, parechovirus/enterovirus; RSV, respiratory syncytial virus.

As seen in Figure 1, the NP/OP specimens detected the majority of infections for most organisms. However, for certain organisms, IS specimens seem superior to NP/OP specimens (eg, in cases with radiographic pneumonia, parainfluenza 1 was detected from IS specimens alone in 2.8% vs 0.5% for NP/OP specimens alone). An analysis stratified by pneumonia severity (severe or very severe) and age <6 months showed pathogen detection rates from NP/OP and IS specimens very similar to those indicated in Table 3 (data not shown). To assess whether the added yield of the IS specimen was related to collection of a sputum specimen per se or to collection of an additional respiratory specimen of any type, we compared the added yield of low-quality IS specimens (ie, those presumably from, or contaminated by, secretions from the upper respiratory tract) with the yield of NP/OP specimens. The added yield of low-quality IS specimens was similar (Figure 1 vs Supplementary Figure 1).

There was no difference in the prevalence or the density of *P. jirovecii* by IS or NP/OP specimens among cases with radiographic pneumonia (Table 3; Supplementary Table 3). Among HIV-positive children with radiographic pneumonia, the overall *P. jirovecii* detection rate was higher (19.7%) than in all HIV-negative cases with radiographic pneumonia (10.6%). In the latter group of HIV-negative children, there was a higher prevalence of *P. jirovecii* (17% vs 9%; *P* = .01) among children who were severely malnourished (weight for age less than −3 z-scores below the median of the WHO child growth standards) than among those who were not. There was little added value in seeking the detection of *P. jirovecii* in IS versus NP/OP specimens. Among 81 HIV-positive children with radiographic pneumonia, *P. jirovecii* was detected in both IS and NP/OP specimens in 13 cases, IS specimens alone in 2, and NP/OP specimens alone in only 1. This represents an increase in yield of only 1% in the detection of *P. jirovecii* when assessing IS specimens in addition to NP/OP specimens (15 of 81 [18.5%] vs 14 of 81 [17.3%]).

Among cases with radiographic pneumonia, pathogen density in either IS or NP/OP specimens did not show a consistent pattern across pathogens. As indicated by the proportion of cases with a difference in PCR density >1 log_10_ copies/mL, the density was greater in the IS specimen than in the NP/OP specimen only for parainfluenza 1, and greater in the NP/OP specimen for 3 organisms (*Haemophilus influenzae*, *M. catarrhalis*, and *S. pneumoniae*) (Supplementary Table 3).

### Viral and Bacterial Organisms Identified by PCR of IS Specimens in Radiographic Pneumonia and Nonpneumonia Case Groups

To assess whether organism detection in IS specimens was associated with radiographic pneumonia, we compared its prevalence in the IS specimens from radiographic pneumonia and nonpneumonia case groups, restricting the analysis to high-quality IS specimens. For 4 organisms (*H. influenzae*, *M. pneumoniae,* parainfluenza 1, and respiratory syncytial virus [RSV]), detection in the IS specimen was associated with radiographic pneumonia case status; however, this association was no longer significant when accounting for the presence of the pathogen in the NP/OP specimen (Table 4). That is to say, no organism was found significantly more frequently in the IS specimen from cases with radiographic pneumonia compared with nonpneumonia cases. However, given the close correlation in presence of organisms between IS and NP/OP specimens (Supplementary Table 2), the presence of the above 4 organisms in either the IS or NP/OP specimen was significantly associated with radiographic pneumonia status compared with nonpneumonia status. An analysis stratified by pneumonia severity (severe and very severe) and age <6 months showed very similar results (Supplementary Tables 4a and 4b).

**Table 4.** PCR Pathogens Detection in High-Quality IS Specimens From Cases With Radiographic Pneumonia or Nonpneumonia

Pathogen	Cases, No. (%)^a^		aOR (95% CI)^b^	
	Radiographic Pneumonia (n = 1166)	Nonpneumonia (n = 398)	Not Adjusted for NP/OP	Adjusted for NP/OP)
*Bordetella pertussis*	10 (0.90)	4 (1.0)	0.66 (.20–2.21)	0.77 (.14–4.21)
*Chlamydophila pneumoniae*	12 (1.1)	4 (1.0)	0.74 (.22–2.42)	0.64 (.18–2.38)
*Haemophilus influenzae*	600 (53.5)	168 (43.2)	1.33 (1.03–1.71)^c^	1.04 (.75–1.45)
*H. influenzae* type b	22 (2.0)	8 (2.1)	0.85 (.36–2.00)	1.07 (.36–3.16)
*Moraxella catarrhalis*	672 (59.9)	258 (66.3)	0.82 (.63–1.07)	0.87 (.62–1.24)
*Mycoplasma pneumoniae*	26 (2.3)	2 (0.5)	5.37 (1.24–23.18)^c^	4.46 (.86–23.08)
*Pneumocystis jirovecii*	94 (8.4)	20 (5.1)	1.24 (.73–2.09)	1.03 (.54–1.98)
*Staphylococcus aureus*	140 (12.5)	46 (11.8)	0.73 (.50–1.08)	0.87 (.55–1.40)
*Streptococcus pneumoniae*	795 (70.9)	279 (71.7)	0.98 (.75–1.29)	0.98 (.66–1.44)
*Salmonella* species	12 (1.1)	5 (1.3)	0.66 (.21–2.03)	0.53 (.16–1.70)
Adenovirus	149 (13.2)	58 (14.8)	0.72 (.51–1.02)	0.74 (.47–1.17)
Bocavirus	160 (14.3)	63 (16.2)	0.85 (.61–1.19)	0.82 (.56–1.21)
CMV	572 (50.8)	204 (52.2)	0.82 (.64, 1.05)	0.69 (.50–.95)^c^
HCoV 229E	14 (1.3)	5 (1.3)	1.03 (.35–2.99)	0.69 (.18–2.66)
HCoV OC43	32 (2.9)	13 (3.3)	0.81 (.40–1.61)	1.24 (.42–3.68)
HCoV NL63	18 (1.6)	12 (3.1)	0.36 (.15–.85)^c^	0.31 (.08–1.20)
HCoV HKU1	23 (2.1)	8 (2.1)	0.84 (.36–2.00)	0.43 (.13–1.38)
HMPV	133 (11.9)	41 (10.5)	1.26 (.85–1.86)	0.71 (.42–1.21)
Influenza A	39 (3.5)	19 (4.9)	0.69 (.38–1.25)	0.48 (.17–1.38)
Influenza B	15 (1.3)	11 (2.8)	0.59 (.26–1.34)	2.13 (.23–20.00)
Influenza C	5 (0.5)	4 (1.0)	0.23 (.06–.95)^c^	0.09 (.01–.99)^c^
Parainfluenza 1	84 (7.5)	18 (4.6)	2.00 (1.14–3.52)^c^	2.17 (.96–4.91)
Parainfluenza 2	19 (1.7)	4 (1.0)	1.83 (.61–5.51)	2.74 (.73–10.31)
Parainfluenza 3	75 (6.7)	21 (5.4)	1.17 (.70–1.97)	1.18 (.53–2.60)
Parainfluenza 4	27 (2.4)	10 (2.6)	0.85 (.39–1.86)	0.52 (.17–1.57)
PV/EV	109 (9.7)	43 (11.0)	0.92 (.62–1.35)	1.03 (.64–1.65)
Rhinovirus	243 (21.7)	92 (23.7)	0.79 (.59–1.05)	0.78 (.54–1.12)
RSV	279 (24.8)	60 (15.3)	2.08 (1.51–2.86)^d^	1.08 (.61–1.89)

Abbreviations: aOR, adjusted odds ratio; CI, confidence interval; CMV, cytomegalovirus; HCoV, human coronavirus; HMPV, human metapneumovirus A/B; IS, induced sputum; NP/OP, nasopharyngeal/oropharyngeal specimen results; PCR, polymerase chain reaction; PV/EV, parechovirus/enterovirus; RSV, respiratory syncytial virus.

^a^Denominators for percentages represent the number of children with available IS specimen results for each pathogen. Some cases had data missing for certain pathogens (<20 cases per pathogen). Radiographic pneumonia was defined as radiographic evidence of pneumonia (consolidation and/or other infiltrates); nonpneumonia, as normal chest radiograph, blood culture negative for pathogens, and either (1) a normal respiratory rate *or* no hypoxemia in the absence of crackles or (2) a normal respiratory rate *and* no hypoxemia in the presence of crackles.

^b^All odds ratios adjusted for age, sex, site, and human immunodeficiency virus status.

^c^ *P* < .05.

^d^ *P* < .002.

**Figure 2.**
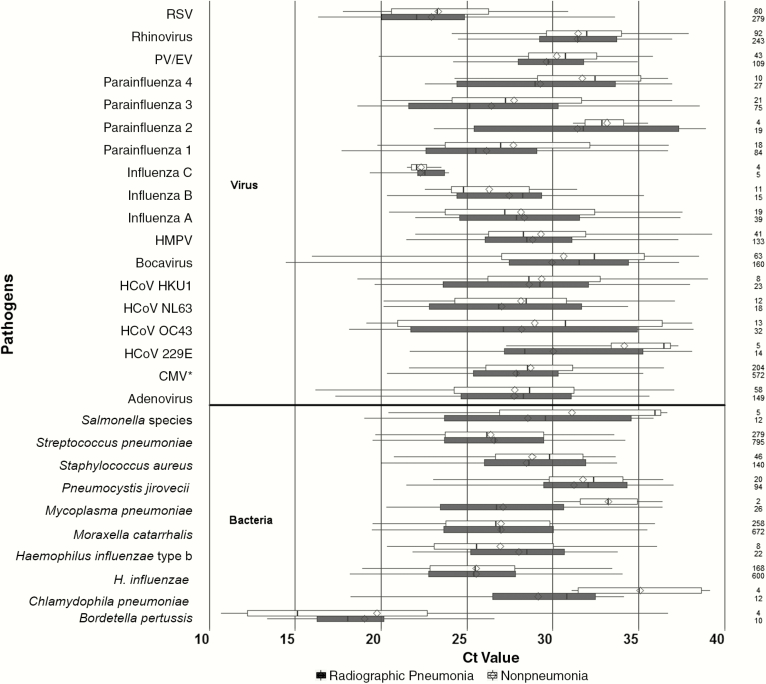
Cycle threshold (Ct) values in induced sputum (IS) specimens of 28 pathogens among cases with radiographic evidence of pneumonia (n = 1166) and those in the nonpneumonia group (n = 398) who had a high-quality IS specimen available. Radiographic evidence of pneumonia was defined as consolidation and/or other infiltrates. Nonpneumonia was defined as a normal chest radiograph, blood culture negative for pathogens, and either (1) a normal respiratory rate *or* no hypoxemia in the absence of crackles or (2) a normal respiratory rate *and* no hypoxemia in the presence of crackles. Diamonds represent group means; boxes, interquartile ranges; vertical lines through boxes, group medians; whiskers, 95% confidence intervals; and numbers on right axis, cases with positive results. *Significant difference (*P* < .05) in density between radiographic pneumonia and nonpneumonia groups. Abbreviations: CMV, cytomegalovirus; HCoV, human coronavirus; HMPV, human metapneumovirus A/B; PV/EV, parechovirus/enterovirus; RSV, respiratory syncytial virus.

To test the hypothesis that organism density should be higher in IS specimens from cases with radiographic pneumonia than nonpneumonia cases if the IS specimen is more closely reflective of the cause of pneumonia, we compared median Ct values for each organism detected with IS specimen PCR. Among high-quality IS specimens, there were no statistically significant differences in organism density in the IS specimens from cases with radiographic pneumonia compared with nonpneumonia cases, with the exception of a marginally significant difference for cytomegalovirus (median Ct, 27.9 vs 28.5; *P* = .01) (Figure 2). Findings were similar among infants aged <6 months (data not shown), with the only difference being greater *S. pneumoniae* density in the nonpneumonia group (median Ct, 27.0 for radiographic pneumonia vs 25.6 for nonpneumonia cases; *P* = .002).

We compared organism detection in the NP/OP specimens between the nonpneumonia cases and the community controls to assess whether our mock control group (ie, nonpneumonia group) was similar to the community control group (Supplementary Table 5). Accounting for multiple comparisons, we found that some pathogens were identified *less* frequently among nonpneumonia cases (mock control group) than among the true controls (adjusted odds ratio for *M. catarrhalis*, 0.63 [95% CI, .51–.78]; *P. jirovecii*, 0.45 [.29–.70]); alternatively, some were more commonly found in nonpneumonia cases (adjusted odds ratio for influenza A, 3.32 [95% CI, 1.93–5.69]; influenza B, 4.07 [CI, 2.14–7.72]; parainfluenza 1, 4.66 [2.83–7.68]; RSV, 5.94 [4.34–8.12]).

## DISCUSSION

Obtaining an uncontaminated diagnostic specimen from the lung in children with pneumonia would significantly enhance clinical case management, as well as our understanding of the microbiological cause of the disease. Although ideal in theory, this is quite difficult to establish in practice. In the current large study of childhood pneumonia etiology, which used highly standardized methods across 9 sites, we found generally good agreement between NP/OP and IS specimens in the detection of common respiratory pathogens with PCR and no clear diagnostic benefit of IS specimens. At a population level, multiplex PCR of IS specimens to identify respiratory organisms is likely to contribute little to our understanding of the etiology of pneumonia, beyond the information provided by NP/OP specimens.

In a recent study reported by Zar et al [32], in which children hospitalized with pneumonia were investigated using both NP and IS specimens and laboratory methods comparable to those used in the PERCH study, a proportion of pneumonia cases were detected in the IS specimens only. We found similar results; however, the fact that the added yield of high-quality IS specimens (ie, presumably those from the lower respiratory tract) was similar to that of low-quality IS specimens (ie, presumably those comprising or contaminated by upper respiratory tract secretions) suggests that some of the “added yield” of IS specimens may be related to collection of an additional specimen from the upper respiratory tract, but not collection of a sputum specimen specifically. We could have more adequately assessed this hypothesis had we collected a second NP/OP specimen and compared results from 1 versus 2 specimens.

Although the prevalence of several organisms was higher in the IS specimens from cases with radiographic pneumonia than in those from the nonpneumonia cases, a similar trend was observed when comparing NP/OP specimens from nonpneumonia cases and controls, so the IS specimen does not add inferential value here. Furthermore, organism detection by IS specimen PCR was not associated with radiographic pneumonia compared with nonpneumonia, after controlling for the organism’s presence in NP/OP specimens. Notably, this comparator group consisted of children who met the WHO case definition of severe pneumonia despite normal chest radiographic findings and the absence of many pneumonia clinical characteristics, so it does not represent a true control group (ie, nonhospitalized children). The observation that the NP/OP specimens from nonpneumonia cases were enriched for certain respiratory pathogens compared with the NP/OP specimens from the controls (Supplemental Table 4) is an important limitation of this analysis and suggests that a proportion of subjects in the nonpneumonia group were hospitalized with clinically milder respiratory illness. An additional limitation to this analysis is that the nonpneumonia group was not equally represented across sites.

The prevalence of individual viral and bacterial organisms detected in IS specimens with molecular methods in the current study is generally similar to that in recent reports by other investigators, although our ability to make comparisons with other studies is limited by differences in case definitions and laboratory methods. In a 2007–2008 study in China of children hospitalized with CAP with pneumonic infiltrates on chest radiographs, a respiratory virus was detected with PCR in 272 of 273 IS specimens from children with CAP (99.6%) and 80 of 81 IS specimens (98.8%) from children with a chronic respiratory condition [17]. The most prevalent viruses in the IS specimens of children with CAP included rhinovirus (17.2%), human bocavirus (28%), RSV (37.4%), cytomegalovirus (92%), and several other human herpesviruses [17]. Although some viruses were detected more frequently in cases with CAP than in those without CAP, NP and OP specimens were not obtained, so it is not known whether the IS specimen would have added diagnostic value above and beyond an NP/OP specimen. In studies in Kenya and Finland, a respiratory pathogen was detected with PCR of IS specimens in more than half of children hospitalized with pneumonia, findings generally similar to those reported here, but with a few notable differences (eg, RSV was detected in 4% of cases in Finland, 16% in Kenya, and 25% in the current study) [13, 14, 16].

A separate analysis of all PERCH cases (not just those with radiographic pneumonia) reports that among 43 cases positive for *Bordetella pertussis* in whom both NP/OP and IS specimens (of any quality) were available, 14 (32.6%) were positive by IS but not NP/OP specimens, 2 (4.7%) were positive by NP/OP but not IS specimens, and 27 (62.8%) were positive by both [33], suggesting that IS specimens contribute substantial yield in the diagnosis of pertussis, a disease not of the lung parenchyma and not typically associated with radiographic changes. The added value in the detection of *P. jirovecii* was minimal in the HIV-positive children with radiographic pneumonia; however, collection of both IS and NP/OP specimens provides the highest yield and may be worthwhile to ensure proper diagnosis and treatment. Determination of infection versus colonization with *P. jirovecii* in these cases would require assessment of organism density and clinical correlation [34]. Although the diagnostic yield from an IS specimen may not be sufficient to justify this mildly invasive procedure as a routine part of the diagnostic workup for CAP, IS specimens are still important in cases with suspected tuberculosis and may also be useful in those in whom certain other pathogens (eg, *B. pertussis, M. pneumoniae*) are suspected. The lack of utility of bacterial cultures of IS specimens in determining the cause of pneumonia in children <5 years of age is reported on elsewhere [20].

Selection of specimens for detecting the potential cause(s) of pneumonia must balance epidemiological sensitivity against the feasibility, costs, and time required for specimen collection and data analysis. Collection of IS specimens is generally well tolerated [31]; however, collection and testing involves significant costs and the impact of the additional detections in determining the cause of pneumonia is minimal for most pathogens.

## Supplementary Data

Supplementary materials are available at *Clinical Infectious Diseases* online. Consisting of data provided by the authors to benefit the reader, the posted materials are not copyedited and are the sole responsibility of the authors, so questions or comments should be addressed to the corresponding author.

## Supplementary Material

Supplemental Tables

## Notes

***Author contributions.*** D. M. T. and L.L.H. led analysis and interpretation and drafted manuscript. W. F., Q. S., and M. L. performed analyses and interpretation of results. P. S., D. E. P., L. M., D. R. F., S. L. Z., S. C. M., C. P., and D. R. M. assisted with interpretation of results and drafting of manuscript. D. M. T., H. C. B., W. A. B., D. R. F., S. R. C. H., O. S. L., M. D. K., K. K. L., S. A. M., K. L. O., J. A. G. S., R.A.K., D.R.M., and L. L. H. conceived and designed the study and supervised study conduct. P. S., L. M., M. A., J. O. A., V. L. B., A. N. D., A. J. D., M. M. H., L. H., Y. J., S. K., D. P. M., S. C. M., O. O., C. P., O. S., P. S., M. S., and M. D. T. were involved in study conduct, data collection and/or data management. All authors reviewed and approved the manuscript. D. M. T. had final responsibility for the decision to submit for publication.

***Acknowledgments.*** We offer sincere thanks to the patients and families who participated in this study. We are grateful to the members of the original study teams (listed below) for conducting the studies and collecting data. We also recognize members of the following groups who contributed to the study design, conduct, interpretation, and/or analysis (see Supplemental Appendix for full list of names): the PERCH Expert Group, the Pneumonia Methods Working Group, the PERCH chest radiograph reading panel, and the PERCH study team contributors. This article is published with the permission of the director of the Kenya Medical Research Institute.

***PERCH Study Group***. Johns Hopkins Bloomberg School of Public Health, Baltimore, Maryland: K. L. O. (principal investigator [PI]), O. S. L. (former PI; current affiliation, Bill & Melinda Gates Foundation, Seattle, Washington**),** M. D. K. (co-PI), D. R. F. (joint affiliation with Centers for Disease Control and Prevention, Atlanta, Georgia), A. N. D., A. J. D., Nicholas Fancourt, W. F., L. L. H., M. M. H., E. Wangeci Kagucia, R. A. K., M. L., D. E. P., C. P., Zhenke Wu, S. L. Z.; the Emmes Corporation, Rockville, Maryland: Nora L. Watson; Nuffield Department of Clinical Medicine, University of Oxford, United Kingdom: Jane Crawley; University of Otago, Christchurch, New Zealand: D. R. M.; icddr,b, Dhaka and Matlab, Bangladesh: W. A. B. (site PI), Hubert P. Endtz, Khalequ Zaman, Doli Goswami, L. H., Y. J., Hasan Ashraf; Medical Research Council, Basse, The Gambia: S. R. C. H. (site PI), Bernard E. Ebruke, M. A., Jessica McLellan, Eunice Machuka, Arifin Shamsul, Syed M. A. Zaman, Grant Mackenzie; KEMRI-Wellcome Trust Research Programme, Kilifi, Kenya: J. A. G. S. (site PI and PERCH co-PI), J. O. A., S. C. M., Alice Kamau, S. K., Micah Silaba Ominde; Division of Infectious Disease and Tropical Pediatrics, Department of Pediatrics, Center for Vaccine Development, Institute of Global Health, University of Maryland School of Medicine, Baltimore, and Centre pour le Développement des Vaccins (CVD-Mali), Bamako, Mali: K. L. K. (site PI), M. D. T., Samba O. Sow, M. S., Boubou Tamboura, Uma Onwuchekwa, Nana Kourouma, Aliou Toure; Respiratory and Meningeal Pathogens Research Unit, University of the Witwatersrand, Johannesburg, South Africa: S. A. M. (site PI), D. P. M., Peter V. Adrian, V. L. B., Locadiah Kuwanda, Azwifarwi Mudau, Michelle J. Groome, Nasreen Mahomed; Thailand Ministry of Public Health–US CDC Collaboration, Nonthaburi: H. C. B. (site PI), Somsak Thamthitiwat, Susan A. Maloney (former site PI), Charatdao Bunthi, Julia Rhodes, P. S., Pasakorn Akarasewi (site co-PI, Ministry of Public Health); Boston University School of Public Health, Massachusetts, and University Teaching Hospital, Lusaka, Zambia: D. M. T. (site PI), L. M., James Chipeta, P. S., James Mwansa, Somwe wa Somwe, Geoffrey Kwenda; Canterbury Health Laboratory, Christchurch, New Zealand: Trevor P. Anderson, Joanne Mitchell.

***Financial support.*** This work is supported by the Bill & Melinda Gates Foundation (grant 48968 to the International Vaccine Access Center, Department of International Health, Johns Hopkins Bloomberg School of Public Health) and the Wellcome Trust of Great Britain (clinical fellowship 098532 to J. A. G. S.).

***Supplement sponsorship.*** This article appears as part of the supplement “Pneumonia Etiology Research for Child Health (PERCH): Foundational Basis for the Primary Etiology Results,” sponsored by a grant from the Bill & Melinda Gates Foundation to the PERCH study of Johns Hopkins Bloomberg School of Public Health, Baltimore, Maryland.

***Potential conflicts of interest.*** M. D. K. has received funding for consultancies from Merck, Pfizer, and Novartis and grant funding from Merck. L. L. H. has received grant funding from Pfizer and GlaxoSmithKline. K. L. K. has received grant funding from Merck Sharp & Dohme. S. A. M. has received honoraria for advisory board participation from the Bill & Melinda Gates Foundation (BMGF), Pfizer, Medimmune, and Novartis, has received institutional grants from GlaxoSmithKline, Novartis, Pfizer, Minervax, and BMGF, and has served on the speakers bureau for Sanofi Pasteur and GlaxoSmithKline. K. L. O. has received grant funding from GSK and Pfizer and participates on technical advisory boards for Merck, Sanofi Pasteur, PATH, Affinivax, and ClearPath. All other authors: No reported conflicts.
