# Supplementary material for: Limited Utility of Polymerase Chain Reaction in Induced Sputum Specimens for Determining the Causes of Childhood Pneumonia in Resource-Poor Settings: Findings From the Pneumonia Etiology Research for Child Health (PERCH) Study
Source: Clin Infect Dis. 2017 May 27;64(Suppl 3):S289–300. doi: 10.1093/cid/cix098 (PMC5447848; doi:10.1093/cid/cix098)
Supplement: Supplemental Tables [file cix098_suppl_Supplemental_Tables.pdf]

Supplemental Tables: Induced Sputum and the Diagnosis of Childhood Pneumonia

Supplementary Table 1: Characteristics of cases with and without induced sputum collection

	Cases without IS Collected N=432	Children with IS Collected N=3800	Total N=4232	p-value[a]
**Characteristic**	n (%)	n (%)		
**Age**				
1-5 months	185 (42.8)	1549 (40.8)	1734 (41.0)	.17
6-11 months	111 (25.7)	860 (22.6)	971 (22.9)	
12-23 months	81 (18.8)	860 (22.6)	941 (22.2)	
24-59 months	55 (12.7)	531 (14.0)	586 (13.8)	
**Very severe pneumonia**	283 (65.5)	1087 (28.6)	1370 (32.4)	<.001
**Female**	222 (51.4)	1594 (41.9)	1816 (42.9)	<.001
**HIV status**				
Positive	46 (10.6)	205 (5.4)	251 (5.9)	<.001
Negative	320 (74.1)	3276 (86.2)	3596 (85.0)	
Unknown	66 (15.3)	319 (8.4)	385 (9.1)	
**Received Oxygen**				
At admission	213 (49.3)	1201 (31.6)	1414 (33.4)	<.001
Ever*	289 (66.9)	1575 (41.4)	1864 (44.0)	<.001
**Died within 30 days of discharge[b]**	231 (58.5)	142 (4.2)	373 (8.8)	<.001

Abbreviations: IS, induced sputum; HIV, human immunodeficiency virus.
[a]P-value obtained from Chi-square test.
[b]Children missing 30-day vital status were assumed to be alive at 30 days.

Supplementary Table 2: Pathogen detection by multiplex PCR of paired nasopharyngeal/oropharyngeal swabs and induced sputum collected from children aged 1-59 months hospitalized cases who did not meet the clinical/laboratory definition for pneumonia[a] (N=398)

Pathogen	NP/OP+ and IS+ N (%)	Only NP/OP+ N (%)	Only IS+ N (%)	P-value[b]
B. pertussis	2 (0.5)	1 (0.3)	2 (0.5)	>.99
C. pneumoniae	1 (0.3)	3 (0.8)	3 (0.8)	>.99
H. influenzae	137 (35.6)	43 (11.2)	30 (7.8)	.16
***H. influenzae* type b**	3 (0.8)	5 (1.3)	4 (1.0)	>.99
M. catarrhalis	241 (62.6)	48 (12.5)	13 (3.4)	< .001**
M. pneumoniae	1 (0.3)	1 (0.3)	1 (0.3)	>.99
Pneumocystis jirovecii	7 (1.8)	8 (2.1)	11 (2.8)	.65
S. aureus	32 (8.3)	25 (6.5)	14 (3.6)	.11
S. pneumoniae	261 (67.8)	31 (8.1)	14 (3.6)	.02
**Salmonella species**	0 (0.0)	2 (0.5)	5 (1.3)	.45
**Adenovirus**	32 (8.3)	14 (3.6)	25 (6.5)	.11
**Bocavirus**	28 (7.3)	17 (4.4)	33 (8.6)	.03
**CMV**	158 (40.9)	33 (8.5)	43 (11.1)	.30
**Coronavirus 229E**	2 (0.5)	1 (0.3)	3 (0.8)	.63
**Coronavirus OC43**	9 (2.3)	2 (0.5)	3 (0.8)	>.99
**Coronavirus NL63**	9 (2.3)	1 (0.3)	3 (0.8)	.63
**Coronavirus HKU1**	4 (1.0)	0 (0.0)	4 (1.0)	.13
**HMPV**	21 (5.5)	2 (0.5)	20 (5.2)	<.001*
**Influenza A**	14 (3.6)	2 (0.5)	5 (1.3)	.45
**Influenza B**	10 (2.6)	1 (0.3)	1 (0.3)	>.99
**Influenza C**	3 (0.8)	0 (0.0)	1 (0.3)	>.99
**Parainfluenza 1**	12 (3.1)	2 (0.5)	6 (1.6)	.29
**Parainfluenza 2**	3 (0.8)	4 (1.0)	1 (0.3)	.38
**Parainfluenza 3**	15 (3.9)	4 (1.0)	5 (1.3)	>.99
**Parainfluenza 4**	7 (1.8)	2 (0.5)	3 (0.8)	>.99
**PV/EV**	25 (6.5)	16 (4.1)	18 (4.7)	.86

**Rhinovirus**	67 (17.4)	24 (6.3)	25 (6.5)	>.99
**RSV**	52 (13.5)	6 (1.6)	7 (1.8)	>.99
**Any bacteria**	348 (89.5)	20 (5.1)	9 (2.3)	.06
**Any virus**	325 (83.5)	16 (4.1)	27 (6.9)	.13
**Any pathogen**	375 (96.4)	7 (1.8)	3 (0.8)	.34

Abbreviations: NP/OP, nasopharyngeal/oropharyngeal; IS, induced sputum; PV/EV, parechovirus/enterovirus; RSV, respiratory syncytial virus; CMV, cytomegalovirus; HMPV, human metapneumovirus A/B.

[a] Non-pneumonia (Non-Pn) case defined as a case with a normal CXR, blood culture pathogen negative, and normal respiratory rate or non-hypoxic in the absence of crackles, or normal respiratory rate and non-hypoxic in the presence of crackles.
[b] P-value obtained by McNemar's Chi-square test.
* Presence in IS alone is significantly greater than NP/OP alone (<.002).
** Presence in NP/OP alone is significantly greater than IS alone (<.002).

Supplementary Figure 1: Pathogen detection by multiplex PCR of paired nasopharyngeal/oropharyngeal swabs and induced sputum collected from children aged 1-59 months hospitalized with CXR+ pneumonia in whom a low quality induced sputum specimens was available

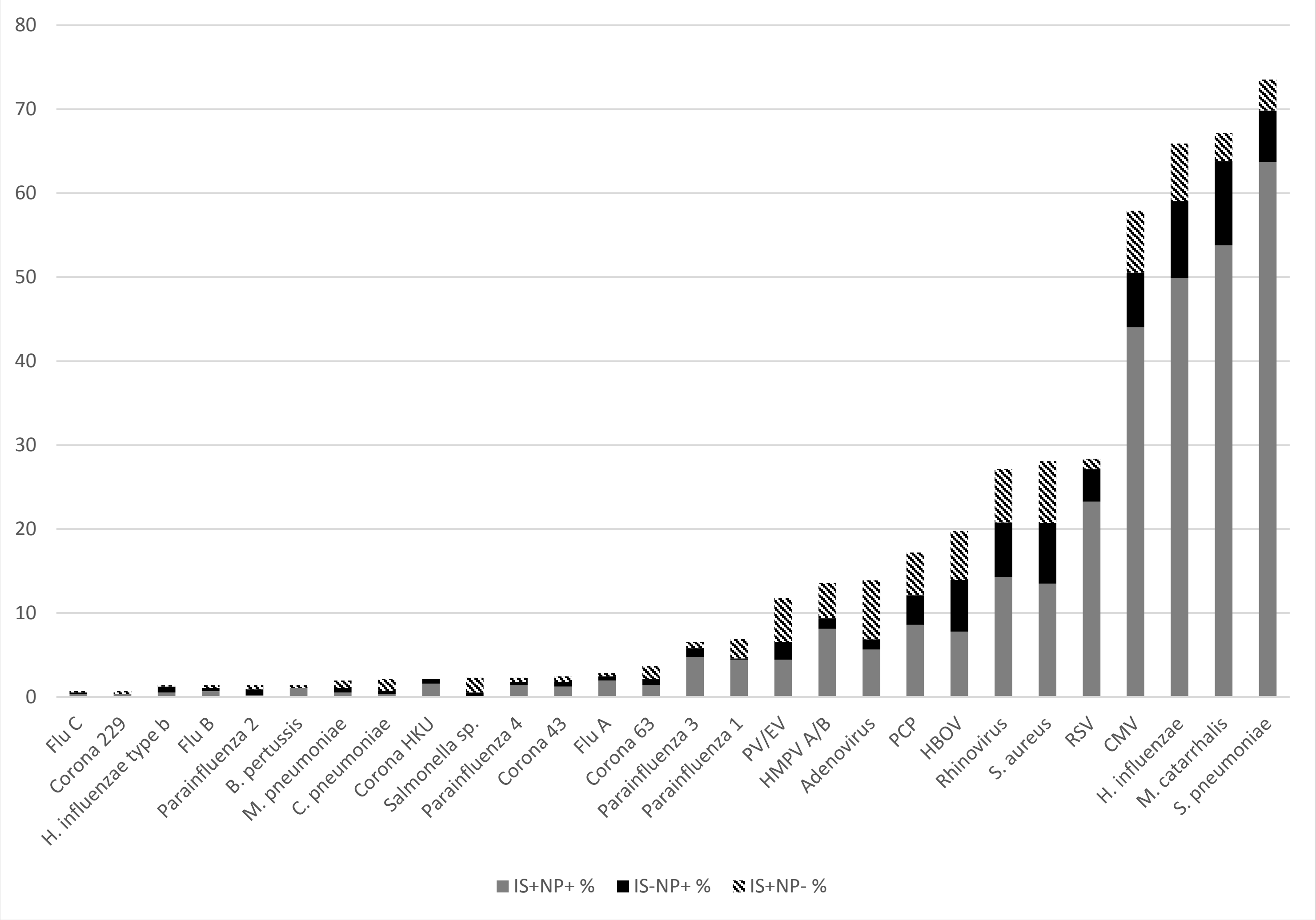

Abbreviations: CXR+, chest x-ray positive; NP, nasopharyngeal/oropharyngeal; IS, induced sputum

Restricted to CXR+ cases with paired NP/OP swabs and low quality IS specimens (≥ 10 squamous epithelial cells per low power field; N=578); some cases missing data for certain pathogens (≤ 20 cases per pathogen). Chest x-ray positive defined as radiographic evidence of pneumonia (consolidation and/or other infiltrates).

Supplementary Table 3: Comparison of pathogen density in induced sputum vs. nasopharyngeal/oropharyngeal specimens among children aged 1-59 months hospitalized with CXR+ pneumonia in whom a high quality induced sputum specimens was available

Pathogen	N	IS greater than NP/OP by 1log (copies/ml) N (%)	NP/OP greater than IS by 1log (copies/ml) N (%)	P-value[a]
B. pertussis	1100	4 (0.4)	5 (0.5)	>.99
C. pneumoniae	1105	7 (0.6)	9 (0.8)	.80
H. influenzae	1105	116 (10.5)	271 (24.5)	**<.001**
***H. influenzae* type b**	1097	7 (0.6)	17 (1.5)	.06
M. catarrhalis	1105	111 (10)	298 (27)	**<.001**
M. pneumoniae	1094	15 (1.4)	5 (0.5)	.04
Pneumocystis jirovecii	1108	34 (3.1)	40 (3.6)	.56
S. aureus	1105	53 (4.8)	85 (7.7)	.008
S. pneumoniae	1105	96 (8.7)	333 (30.1)	**<.001**
***Salmonella* species**	1108	10 (0.9)	9 (0.8)	>.99
**Adenovirus**	1097	70 (6.4)	38 (3.5)	.003
**Bocavirus**	1094	103 (9.4)	79 (7.2)	.09
**CMV**	1097	127 (11.6)	180 (16.4)	.003
**Coronavirus 229E**	1094	7 (0.6)	7 (0.6)	>.99
**Coronavirus OC43**	1094	18 (1.6)	6 (0.5)	.002
**Coronavirus NL63**	1094	6 (0.5)	6 (0.5)	>.99
**Coronavirus HKU1**	1094	8 (0.7)	12 (1.1)	.50
**HMPV**	1094	47 (4.3)	39 (3.6)	.45
**Influenza A**	1094	10 (0.9)	11 (1)	>.99
**Influenza B**	1094	6 (0.5)	1 (0.1)	.13
**Influenza C**	1105	1 (0.1)	4 (0.4)	.38
**Parainfluenza 1**	1094	42 (3.8)	13 (1.2)	**<.001**
**Parainfluenza 2**	1096	12 (1.1)	7 (0.6)	.36
**Parainfluenza 3**	1096	23 (2.1)	23 (2.1)	>.99
**Parainfluenza 4**	1096	13 (1.2)	14 (1.3)	>.99
**PV/EV**	1097	64 (5.8)	41 (3.7)	.03
**Rhinovirus**	1094	99 (9)	92 (8.4)	.66

**RSV**	1097	57 (5.2)	84 (7.7)	.03

Abbreviation: CXR+. Chest x-ray positive; IS, induced sputum; NP/OP, nasopharyngeal/oropharyngeal; PV/EV, parechovirus/enterovirus; RSV, respiratory syncytial virus; CMV, cytomegalovirus; HMPV, human metapneumovirus A/B.

Restricted to Chest x-ray positive cases with paired NP/OP swabs and high quality IS specimens (< 10 squamous epithelial cells per low power field; N=1114); some cases missing data for certain pathogens (≤ 20 cases per pathogen). Chest x-ray positive defined as radiographic evidence of pneumonia (consolidation and/or other infiltrates).

Bold indicates significant difference in proportion of children with IS density greater than or less than 1 log more than NP/OP density ($p < 0.002$).

[a] P-value obtained by two-sided sign test.

Supplementary Table 4a. Pathogens detected by PCR in Induced Sputum (IS) among Chest X-Ray Positive Pneumonia Cases and Non-Pneumonia Cases with a high quality IS specimen, Stratified by age

	**Age < 6 months**				**Age > 6 months**			
**Pathogen**	CXR+ (N=418) n (%)	Non-Pneumonia cases (N=145) n (%)	aOR (95% CI)	aOR (also adjusted for NP/OP) (95% CI)	CXR+ (N=700) n (%)	Non-Pneumonia cases (N=243) (%)	aOR (95% CI)	aOR (also adjusted for NP/OP) (95% CI)
B. pertussis	8 (1.9)	3 (2.1)	0.84 (0.21, 3.33)	1.2 (0.16, 9.09)	2 (0.3)	1 (0.4)	0.28 (0.02, 3.43)	0.16 (0.01, 2.88)
C. pneumoniae	4 (1)	2 (1.4)	0.57 (0.1, 3.29)	0.55 (0.06, 4.83)	8 (1.1)	2 (0.8)	0.92 (0.18, 4.76)	0.83 (0.15, 4.62)
H. influenzae	213 (50.7)	65 (44.5)	1.3 (0.86, 1.96)	1.06 (0.58, 1.92)	387 (55.1)	103 (42.4)	1.32 (0.95, 1.83)	1.06 (0.71, 1.57)
***H. influenzae* type b**	10 (2.4)	3 (2.1)	1.16 (0.3, 4.48)	1.82 (0.27, 12.41)	12 (1.7)	5 (2.1)	0.68 (0.22, 2.13)	0.7 (0.18, 2.65)
M. catarrhalis	229 (54.5)	94 (64.4)	0.74 (0.49, 1.13)	0.93 (0.53, 1.62)	443 (63.1)	164 (67.5)	0.85 (0.6, 1.19)	0.86 (0.55, 1.34)
M. pneumoniae	5 (1.2)	0 (0)	--	--	21 (3)	2 (0.8)	4.9 (1.11, 21.75)*	4.77 (0.78, 28.98)
Pneumocystis jirovecii	70 (16.7)	19 (13)	1.15 (0.64, 2.04)	0.75 (0.37, 1.52)	24 (3.4)	1 (0.4)	5.5 (0.71, 42.84)	--
S. aureus	77 (18.3)	32 (21.9)	0.63 (0.38, 1.04)	0.71 (0.38, 1.32)	63 (9)	14 (5.8)	1.03 (0.53, 1.99)	1.19 (0.56, 2.51)
S. pneumoniae	277 (66)	97 (66.4)	0.99 (0.65, 1.51)	0.99 (0.52, 1.87)	518 (73.8)	182 (74.9)	0.93 (0.64, 1.34)	0.99 (0.61, 1.63)
**Salmonella species**	5 (1.2)	1 (0.7)	1.46 (0.16, 13.52)	1.02 (0.08, 13.14)	7 (1)	4 (1.6)	0.46 (0.11, 1.82)	0.46 (0.12, 1.85)
**Adenovirus**	19 (4.5)	8 (5.5)	0.62 (0.25, 1.53)	0.67 (0.22, 2.06)	130 (18.5)	50 (20.4)	0.68 (0.46, 1.02)	0.68 (0.41, 1.13)
**Bocavirus**	22 (5.2)	16 (11)	0.45 (0.22, 0.92)*	0.34 (0.14, 0.79)*	138 (19.7)	47 (19.3)	0.98 (0.66, 1.45)	1 (0.64, 1.54)
**CMV**	172 (40.9)	57 (39)	0.97 (0.64, 1.47)	0.83 (0.43, 1.6)	400 (56.8)	147 (60)	0.71 (0.51, 0.99)*	0.65 (0.44, 0.95)*
**Coronavirus 229E**	7 (1.7)	1 (0.7)	2.54 (0.3, 21.65)	2.2 (0.17, 28.78)	7 (1)	4 (1.7)	0.61 (0.16, 2.25)	0.35 (0.06, 2.09)
**Coronavirus OC43**	13 (3.1)	3 (2.1)	1.29 (0.35, 4.84)	--	19 (2.7)	10 (4.1)	0.64 (0.28, 1.49)	0.97 (0.3, 3.18)
**Coronavirus NL63**	6 (1.4)	4 (2.7)	0.32 (0.08, 1.29)	--	12 (1.7)	8 (3.3)	0.38 (0.13, 1.14)	0.33 (0.07, 1.55)
**Coronavirus HKU1**	11 (2.6)	3 (2.1)	1.03 (0.27, 4)	0.52 (0.1, 2.72)	12 (1.7)	5 (2.1)	0.68 (0.22, 2.15)	0.37 (0.06, 2.12)

**HMPV**	37 (8.8)	19 (13)	0.75 (0.41, 1.39)	0.31 (0.12, 0.77)*	96 (13.7)	22 (9.1)	1.64 (0.97, 2.79)	1.13 (0.58, 2.21)
**Influenza A**	12 (2.9)	3 (2.1)	1.51 (0.41, 5.51)	0.67 (0.13, 3.53)	27 (3.9)	16 (6.6)	0.5 (0.25, 1.01)	0.32 (0.08, 1.34)
**Influenza B**	5 (1.2)	4 (2.7)	0.5 (0.13, 1.95)	--	10 (1.4)	7 (2.9)	0.65 (0.23, 1.83)	1.7 (0.14, 20.96)
**Influenza C**	4 (1)	1 (0.7)	0.8 (0.08, 7.84)	--	1 (0.1)	3 (1.2)	0.07 (0.01, 0.7)*	0.05 (0, 1.02)
**Parainfluenza 1**	35 (8.3)	9 (6.2)	1.92 (0.81, 4.56)	2.31 (0.73, 7.29)	49 (7)	9 (3.7)	2.31 (1.07, 4*.99)	2.3 (0.71, 7.43)
**Parainfluenza 2**	5 (1.2)	1 (0.7)	2.03 (0.23, 18.03)	4.76 (0.4, 56.62)	14 (2)	3 (1.2)	1.71 (0.47, 6.24)	1.54 (0.33, 7.19)
**Parainfluenza 3**	26 (6.2)	6 (4.1)	1.38 (0.54, 3.51)	1.07 (0.29, 4)	49 (7)	15 (6.1)	1.09 (0.57, 2.06)	1.34 (0.48, 3.7)
**Parainfluenza 4**	8 (1.9)	5 (3.4)	0.55 (0.17, 1.8)	--	19 (2.7)	5 (2)	1.14 (0.39, 3.37)	0.68 (0.19, 2.43)
**PV/EV**	25 (5.9)	16 (11)	0.47 (0.24, 0.95)*	0.58 (0.24, 1.4)	84 (11.9)	27 (11)	1.22 (0.75, 1.99)	1.35 (0.76, 2.39)
**Rhinovirus**	89 (21.1)	21 (14.4)	1.46 (0.86, 2.5)	1.32 (0.69, 2.51)	154 (22)	71 (29.2)	0.54 (0.38, 0.78)*	0.56 (0.35, 0.88)*
**RSV**	141 (33.5)	45 (30.8)	1.32 (0.86, 2.04)	0.88 (0.39, 1.97)	138 (19.6)	15 (6.1)	4.39 (2.47, 7.8)*	1.52 (0.65, 3.58)

Abbreviations: aOR, adjusted odds ratio; CXR+, chest x-ray positive; PV/EV, parechovirus/enterovirus; RSV, respiratory syncytial virus; CMV, cytomegalovirus; HMPV, human metapneumovirus A/B.

The denominator was the number of children with available IS results for each pathogen. Some cases missing data for certain pathogens (<20 cases per pathogen).
CXR+ defined as radiographic evidence of pneumonia (consolidation and/or other infiltrates).
Non-pneumonia (Non-Pn) case defined as a case with a normal CXR, blood culture pathogen negative, and normal respiratory rate or non-hypoxic in the absence of crackles, or normal respiratory rate and non-hypoxic in the presence of crackles.
OR adjusted for age, sex, site and HIV status.
*p<.05 and **p<.002.

Supplementary Table 4b. Pathogens detected by PCR in Induced Sputum (IS) among Chest X-Ray Positive Pneumonia Cases and Non-Pneumonia Cases with a high quality IS specimen, Stratified by severity

	Severe				Very Severe			
**Pathogen**	CXR+ (N=829) n (%)	Non-Pneumonia cases (N=236) n (%)	aOR (95% CI)	aOR (also adjusted for NP/OP) (95% CI)	CXR+ (N=289) n (%)	Non-Pneumonia cases (N=152) n (%)	aOR (95% CI)	aOR (also adjusted for NP/OP) (95% CI)
B. pertussis	6 (0.7)	3 (1.3)	0.52 (0.12, 2.24)	0.51 (0.08, 3.16)	4 (1.4)	1 (0.7)	0.69 (0.07, 7)	--
C. pneumoniae	9 (1.1)	3 (1.3)	0.68 (0.17, 2.74)	0.57 (0.12, 2.79)	3 (1)	1 (0.7)	1.05 (0.08, 13.25)	1.05 (0.08, 14.7)
H. influenzae	455 (54.6)	102 (43.4)	1.59 (1.15, 2.19)*	1.38 (0.91, 2.09)	145 (50.3)	66 (42.9)	0.91 (0.57, 1.46)	0.6 (0.33, 1.08)
***H. influenzae* type b**	13 (1.6)	3 (1.3)	1.16 (0.31, 4.31)	1.54 (0.37, 6.51)	9 (3.1)	5 (3.3)	0.73 (0.19, 2.75)	--
M. catarrhalis	507 (60.8)	156 (66.4)	0.83 (0.59, 1.15)	0.75 (0.48, 1.18)	0 (0)	1 (0.8)	0.75 (0.46, 1.2)	1.06 (0.58, 1.92)
M. pneumoniae	23 (2.8)	0 (0)	--	--	165 (57.3)	102 (66.2)	1.03 (0.15, 6.97)	0.96 (0.11, 8.52)
Pneumocystis jirovecii	66 (7.9)	16 (6.8)	1.22 (0.66, 2.25)	1.04 (0.5, 2.17)	5 (1.7)	3 (2)	1.62 (0.49, 5.32)	1.33 (0.28, 6.39)
S. aureus	103 (12.4)	38 (16.2)	0.63 (0.4, 0.99)*	0.81 (0.48, 1.4)	2 (0.7)	2 (1.3)	1.58 (0.61, 4.08)	1.73 (0.57, 5.2)
S. pneumoniae	591 (70.9)	166 (70.6)	1.01 (0.72, 1.42)	1.01 (0.61, 1.66)	28 (9.7)	4 (2.6)	0.94 (0.56, 1.57)	0.89 (0.45, 1.78)
**Salmonella species**	10 (1.2)	3 (1.3)	0.68 (0.16, 2.8)	0.64 (0.15, 2.66)	70 (24.3)	13 (8.4)	0.38 (0.04, 3.54)	0.19 (0.01, 3.01)
**Adenovirus**	113 (13.5)	34 (14.3)	0.68 (0.43, 1.06)	0.68 (0.38, 1.21)	36 (12.5)	24 (15.6)	0.72 (0.37, 1.41)	0.94 (0.41, 2.13)
**Bocavirus**	132 (15.8)	40 (17)	0.77 (0.5, 1.17)	0.79 (0.49, 1.27)	28 (9.7)	23 (14.9)	0.74 (0.37, 1.48)	0.69 (0.32, 1.45)
**CMV**	433 (51.7)	114 (48.1)	0.96 (0.7, 1.32)	0.88 (0.58, 1.33)	139 (48.3)	90 (58.4)	0.54 (0.33, 0.87)*	0.49 (0.27, 0.86)*
**Coronavirus 229E**	10 (1.2)	4 (1.7)	0.71 (0.21, 2.41)	0.39 (0.08, 1.89)	4 (1.4)	1 (0.7)	1.67 (0.17, 16.1)	1.93 (0.12, 31.88)
**Coronavirus OC43**	24 (2.9)	6 (2.6)	1.05 (0.41, 2.69)	1.47 (0.38, 5.63)	8 (2.8)	7 (4.6)	0.55 (0.17, 1.77)	1.98 (0.16, 24.45)
**Coronavirus NL63**	15 (1.8)	5 (2.1)	0.48 (0.15, 1.49)	0.38 (0.06, 2.19)	3 (1)	7 (4.6)	0.34 (0.08, 1.49)	0.34 (0.03, 4.53)
**Coronavirus HKU1**	18 (2.2)	4 (1.7)	1.13 (0.36, 3.53)	0.48 (0.11, 2.05)	5 (1.7)	4 (2.6)	0.49 (0.11, 2.16)	0.37 (0.05, 2.85)
**HMPV**	107 (12.8)	32 (13.6)	1.03 (0.66, 1.63)	0.65 (0.36, 1.19)	26 (9)	9 (5.8)	1.72 (0.72, 4.13)	0.89 (0.26, 3.04)
**Influenza A**	28 (3.4)	7 (3)	1.07 (0.45, 2.55)	0.86 (0.18, 4.22)	11 (3.8)	12 (7.8)	0.47 (0.17, 1.29)	0.32 (0.07, 1.48)
**Influenza B**	11 (1.3)	4 (1.7)	0.84 (0.25, 2.79)	--	4 (1.4)	7 (4.6)	0.52 (0.13, 1.97)	1.34 (0.11, 15.77)
**Influenza C**	3 (0.4)	2 (0.9)	0.31 (0.05, 2.11)	--	2 (0.7)	2 (1.3)	0.11 (0.01, 1.08)	0.12 (0.01, 2.99)
**Parainfluenza 1**	72 (8.6)	16 (6.8)	1.89 (1.01, 3.54)*	2.22 (0.94, 5.2)	3 (1)	2 (1.3)	4.6 (0.91, 23.34)	2.56 (0.11, 57.84)
**Parainfluenza 2**	14 (1.7)	2 (0.8)	2.25 (0.5, 10.11)	3.8 (0.65, 22.33)	12 (4.2)	2 (1.3)	0.87 (0.14, 5.38)	1.99 (0.2, 19.89)
**Parainfluenza 3**	60 (7.2)	15 (6.3)	1.01 (0.55, 1.85)	1.04 (0.4, 2.71)	5 (1.7)	2 (1.3)	1.45 (0.48, 4.32)	1.34 (0.3, 6.01)

**Parainfluenza 4**	22 (2.6)	7 (3)	0.86 (0.35, 2.13)	0.48 (0.13, 1.76)	15 (5.2)	6 (3.9)	0.8 (0.15, 4.29)	0.23 (0.02, 2.6)
**PV/EV**	76 (9.1)	29 (12.2)	0.67 (0.41, 1.07)	0.71 (0.41, 1.24)	204 (70.8)	113 (73.4)	1.71 (0.83, 3.53)	3.25 (1.24, 8.49)*
**Rhinovirus**	172 (20.6)	55 (23.4)	0.74 (0.51, 1.06)	0.86 (0.54, 1.37)	33 (11.5)	14 (9.1)	0.9 (0.53, 1.52)	0.65 (0.33, 1.27)
**RSV**	209 (25)	47 (19.8)	1.82 (1.23, 2.67)*	0.81 (0.42, 1.53)	71 (24.7)	37 (24)	2.6 (1.31, 5.18)*	1.44 (0.4, 5.25)

Abbreviations: aOR, adjusted odds ratio; CXR+, chest x-ray positive; PV/EV, parechovirus/enterovirus; RSV, respiratory syncytial virus; CMV, cytomegalovirus; HMPV, human metapneumovirus.

The denominator was the number of children with available IS results for each pathogen. Some cases missing data for certain pathogens (<20 cases per pathogen).
CXR+ defined as radiographic evidence of pneumonia (consolidation and/or other infiltrates).
Non-pneumonia (Non-Pn) case defined as a case with a normal CXR, blood culture pathogen negative, and normal respiratory rate or non-hypoxic in the absence of crackles, or normal respiratory rate and non-hypoxic in the presence of crackles.
OR adjusted for age, sex, site and HIV status.
*p<.05 and **p<.002.

Supplemental Tables: Induced Sputum and the Diagnosis of Childhood Pneumonia

Supplementary Table 5. Pathogens detected by PCR in nasopharyngeal/oropharyngeal specimens from non-pneumonia cases and controls

**Pathogen**	**Non-Pn Cases N=573**	**Controls N=5325**	**Non-Pn Cases vs. Controls**	
	n (%)	n (%)	aOR (95% CI)	P-Value
B. pertussis	3 (0.5)	11 (0.2)	3.04 (0.79, 11.69)	.11
C. pneumoniae	5 (0.9)	66 (1.3)	0.64 (0.25, 1.63)	.35
H. influenzae	277 (48.9)	2653 (51.2)	0.96 (0.80, 1.16)	.67
***H. influenzae* type b**	12 (2.1)	89 (1.7)	1.37 (0.73, 2.58)	.33
M. catarrhalis	407 (71.9)	3812 (73.5)	0.63 (0.51, 0.78)	<.001**
M. pneumoniae	4 (0.7)	71 (1.4)	0.58 (0.21, 1.63)	.30
Pneumocystis jirovecii	24 (4.2)	395 (7.6)	0.45 (0.29, 0.70)	<.001**
S. aureus	87 (15.4)	711 (13.7)	1.43 (1.11, 1.85)	.006
S. pneumoniae	421 (74.4)	3973 (76.6)	0.76 (0.62, 0.94)	.01
***Salmonella* species**	2 (0.4)	27 (0.5)	0.52 (0.12, 2.22)	.38
**Adenovirus**	65 (11.5)	609 (11.7)	1.32 (0.99, 1.76)	.06
**Bocavirus**	61 (10.8)	684 (13.2)	0.87 (0.65, 1.15)	.33
**CMV**	273 (48.3)	2787 (53.7)	0.90 (0.75, 1.08)	.27
**Coronavirus 229E**	5 (0.9)	55 (1.1)	0.79 (0.31, 2.03)	.63
**Coronavirus OC43**	13 (2.3)	204 (3.9)	0.57 (0.32, 1.02)	.06
**Coronavirus NL63**	16 (2.8)	161 (3.1)	0.89 (0.52, 1.51)	.66
**Coronavirus HKU1**	7 (1.2)	117 (2.3)	0.54 (0.25, 1.17)	.12
**HMPV**	41 (7.3)	212 (4.1)	1.63 (1.13, 2.35)	.01
**Influenza A**	20 (3.5)	59 (1.1)	3.32 (1.93, 5.69)	<.001*
**Influenza B**	16 (2.8)	31 (0.6)	4.07 (2.14, 7.72)	<.001*
**Influenza C**	3 (0.5)	31 (0.6)	0.79 (0.23, 2.66)	.70

**Parainfluenza 1**	28 (5.0)	52 (1.0)	4.66 (2.83, 7.68)	<.001*
**Parainfluenza 2**	11 (2.0)	56 (1.1)	1.85 (0.94, 3.67)	.08
**Parainfluenza 3**	29 (5.1)	148 (2.9)	1.86 (1.22, 2.85)	.004
**Parainfluenza 4**	13 (2.3)	88 (1.7)	1.45 (0.79, 2.67)	.23
**PV/EV**	54 (9.6)	436 (8.4)	1.16 (0.85, 1.58)	.35
**Rhinovirus**	136 (24.1)	1088 (21.0)	1.17 (0.95, 1.45)	.14
**RSV**	78 (13.8)	145 (2.8)	5.94 (4.34, 8.12)	<.001*

Abbreviation: aOR, adjusted odds ratio; CI, confidence interval.
OR adjusted for age, sex, site and HIV status.
Non-pneumonia (Non-Pn) case defined as a case with a normal CXR, blood culture pathogen negative, and normal respiratory rate or non-hypoxemic in the absence of crackles, or normal respiratory rate and non-hypoxemic in the presence of crackles.
* Presence in Non-Pn cases is significantly greater than in controls (<.002).
** Presence in controls is significantly greater than in Non-Pn cases (<.002).

Supplemental Tables: Induced Sputum and the Diagnosis of Childhood Pneumonia

Acknowledgements:

PERCH Expert Group. William C. Blackwelder, Harry Campbell, John A. Crump, Adegoke Falade, Menno D. de Jong, Claudio Lanata, Kim Mulholland, Shamim Qazi, Cynthia G. Whitney.

Pneumonia Methods Working Group. Robert E Black, Zulfiqar A Bhutta, Harry Campbell, Thomas Cherian, Derrick W Crook, Menno D de Jong, Scott F Dowell, Stephen M Graham, Keith P Klugman, Claudio F Lanata, Shabir A Madhi, Paul Martin, James P Nataro, Franco M Piazza, Shamim A Qazi, and Heather J Zar.

PERCH Chest Radiograph Reading Panel

Readers: Dr. Kamrun Nahar, Dr. Fariha Bushra Matin, Dr. Claire Oluwalana, Dr. Bernard E. Ebruke, Dr. Joyce Sande, Dr. Micah Silaba Ominde, Dr. Mahamadou Diallo, Dr. Breanna Barger-Kamate, Dr. Nasreen Mahomed, Dr. David P. Moore, Dr. Anchalee Kruatrachue, Dr. Piyarat Suntarattiwong, Dr. Musaku Mwenechanya, Dr. Rasa Izadnegahdar, **Arbitrators:** Dr. Vera Manduku, Dr. John DeCampo, Dr. Marg DeCampo, Dr. Fergus Gleeson.

PERCH Contributors:

Bangladesh: Kamrun Nahar, Arif Uddin Sikdir, Sharifa Yeasmin, Dilruba Ahmed, Muhammad Ziaur Rahman, Muhammad Yunus, Muhammad Al Fazl Khan, Muhammad Jubayer Chisti, Abu Sadat Muhammad Sayeem, Shahriar Bin Elahi, Mustafizur Rahman; **The Gambia:** Michel Dione, Emmanuel Olutunde, Peter Githua, Ogochukwu Ofordile, Rasheed Salaudeen, David Parker; **Kenya:** Shebe Mohamed, Siti Ndaa, Micah Silaba, Neema Muturi, Angela Karani, Sammy Nyongesa, Anne Bett, Daisy Mugo, Salim Mwarumba, Robert Musyimi, Andrew Brent, James Nokes, David Mulewa, Joyce Sande, John Odhiambo, Joshua Wambua, Nuru Kibirige, Caroline Mulunda, Hellen Mjalla, Norbert Katira, Karen Dama, Loice Masha, Christine Mutunga, Mwanajuma Ngama, Stephen Mangi, Riziki Anthony, Mwarua Yubu, Elijah Wakili, Benson Katana, Shoboi Mgunya, Emmanuel Mumba, Benedict Mver, George Kuria, Felix Githinji, Norbert Kihuha, Boniface Jibendi, Tahreni Bwanaali, Agustus Kea; **Mali:** Nana Kourouma, Aliou Toure, Mahamadou Diallo, Breana Barger-Kamate, Mariam Samake, Seydou Sissoko, Abdoul Aziz Maiga, Mariam Samake, Toumani Sidibe, Mariam Sylla, Aziz Diakite, Bassirou Diarra; **South Africa:** Azwidihwi Takalani, Andrea Hugo, Susan Nzenze, Ndulela Titi, Mmabatho Selela, Malebo Motiane, Minah Nkuna, Nonhlanhla Tsholetsane, Sibonsile Moya, Debra Katisi, Tondani Netshishivhe, Lerato Mapetla, Gudani Singo, Simphiwe Gasa, Cece Mgenge, Nozipho Mthunzi, Nombulelo Monedi, Tanja Adams, Shafeeka Mangera, Jeannette Wadula, Peter Tsaagane, Jenifer L. Vaughan, Sakina Loonat, Martin Hale, Sugeshnee Pather, Mariëtte Middel, Siobhan Trenor, Palesa

Morailane, Ntombi Maya, Rene Sterley, Charné Combrinck, Given Malete, Lerato Qoza, Grizelda Liebenberg, Hendrik van Jaarsveld, Zunaid Kraft, Lisa-Marie Mollentze, Lourens Combrinck, Tsholofelo Mosome; **Thailand:** Sununta Henchaichon, Dr. Tussanee Amornintapichet, Dr. Somchai Chuananont, Toni Whistler, Juraiporn Ratanodom, Patranuch Sapchookul, Ornuma Sangwichian, Sirirat Makprasert, Manoon Hirunsalee, Possawat Jorakate, Anek Kaewpan, Duangkamol Siludjai, Apiwat Lapamnouysup, Dr. Wantana Paveenkittiporn, Waraporn Ubonphen, Dr. Peera Areerat, Ms.Yupapan Wannachaiwong, Ms Tewa Faipet, Ms Punnat Natnarakorn, Ms Ahchanan Sacharone, Mr.Winai Makmool, Ms. Kanlaya Sornwong, Ms. Promporn Sansuriwong, Ms. Ratchanida Potiya, Ms. Wasana Hongsawong, Ms.Wipa Matchaikhen, Ms. Thatsanawan Chaiyabil, Ms.Piyapai Wannarach, Ms Chamaiporn Wadeesirisak, Mr. Yuttapong Norapet, Mattana Bangkung, Mr. Barameht Piralam, Sathapana Naorat, Anchalee Jatapai, Prasong Srisaengchai, Dr. Leonard Peruski, Ms.Dawan Phaensoongnoen, Ms.Tussaaorn Klangprapan, Ms.Narawadee Dumrongdee, Ms.Atchara Srithongkham, Mr. Piyawut Noinont, Ms. Pornthip Kamlee, Ms.Siyapa Mongkornsuk; **Zambia:** Justin Mulindwa, Musaku Mwenechanya, John Mwaba, Magdalene Mwale, Julie Duncan, Kazungu Siazele, Muntanga Mapeni, Emily Hammond; **Canterbury Health Laboratory, Christchurch, New Zealand:** Rose Watt, Shalika Jayawardena; **The Emmes Corporation, Rockville, Maryland:** Mark Wolff, Megan Sanza, Omid Neyzari.
